# Control of renal calcium permeability via a tight junctional claudin switch

**DOI:** 10.1073/pnas.2512046122

**Published:** 2025-12-01

**Authors:** Rozemarijn E. van der Veen, Marie Bieck, Nacéra Mezouar, Volker Haucke, Henrik Dimke, Martin Lehmann

**Affiliations:** ^a^Department of Molecular Physiology and Cell Biology, Leibniz Forschungsinstitut für Molekulare Pharmakologie, Berlin 13125, Germany; ^b^Faculty of Biology, Chemistry, Pharmacy, Freie Universität Berlin, Berlin 14195, Germany; ^c^Charité Universitätsmedizin Berlin, Corporate Member of Freie Universität Berlin, and Humboldt-Universität zu Berlin, and Berlin Institute of Health, NeuroCure Cluster of Excellence, Berlin 10117, Germany; ^d^Department of Cardiovascular and Renal Research, Institute of Molecular Medicine, University of Southern Denmark, Odense C 5000, Denmark; ^e^Department of Nephrology, Odense University Hospital, Odense C 5000, Denmark

**Keywords:** Claudins, kidney stone disease, paracellular calcium transport, super-resolution microscopy, tight junction

## Abstract

Tight junctions seal the paracellular space between epithelial cells, with their claudin (CLDN) composition dictating epithelial permeability properties. In kidney thick ascending limbs, calcium is reabsorbed paracellularly through a meshwork of CLDN16 and CLDN19 polymers. CLDN14 is strongly upregulated by high blood calcium, restricts this paracellular calcium flux, and is linked to kidney stone disease. How CLDN14 controls paracellular calcium flux and structurally incorporates into this complex junction is unknown. Using confocal and super-resolution microscopy, we show that CLDN14 preferentially associates with CLDN19, thereby gradually replacing CLDN16 in the CLDN19 copolymer in vitro and in mice in vivo. The claudin switch depends on CLDN14 polymerization and occurs on a timescale of days via a pathway independent of dynamin-mediated endocytosis. Our findings reveal a mechanism of tight junction regulation, demonstrating how dynamic claudin remodeling within this complex structure controls renal calcium excretion and contributes to kidney stone pathogenesis.

## Significance

Regulation of calcium transport in the kidney is critical to maintain a systemic calcium balance in the human body. Claudin (CLDN) proteins are found in tight junctions between epithelial cells and form highly polymerized structures. In the kidney, CLDN16 and CLDN19 form a polymer that is permeable to calcium. CLDN14 can restrict paracellular calcium flux and is associated with kidney stone formation. How CLDN14 integrates into the polymer and blocks calcium flux is not known. Using cell and mouse models, we show that CLDN14 acts by switching out CLDN16 to block permeation through the tight junction. This mechanism demonstrates how dynamic claudin remodeling can control renal calcium reabsorption and contribute to kidney stone formation.

To ensure survival, multicellular organisms depend on the compartmentalization of tissues, a function carried out by epithelial cells and their tight junctions (TJs). TJs are specialized multiprotein complexes composed of transmembrane proteins and other constituents that anchor them to the cytoskeleton. The permeability characteristics of the paracellular pathway are primarily regulated by claudins (1, 2). These four-span membrane proteins reside in the TJ and generate specialized permeability profiles across epithelia by conferring either barrier- or pore-forming properties (3–5).

The presence of pore-forming claudins, such as CLDN2, CLDN10, CLDN15, and CLDN16, is critical for the paracellular transport of a variety of ions and water in epithelia with high transport activity, like the intestine and kidney (6, 7). The kidney plays an important role in maintaining calcium (Ca^2+^) balance by regulating the excretion of Ca^2+^ into urine, in response to elevated blood Ca^2+^ levels. Transport of Ca^2+^ in the thick ascending limb (TAL), a tubular segment of the kidney nephron, is responsible for reclaiming as much as 20% of filtered Ca^2+^ (8). Ca^2+^ transport in this segment is largely paracellular and driven by a lumen-positive voltage gradient (9). CLDN16 and CLDN19 colocalize at the TJ in a subset of TAL cells (10–12), where they permit the transport of the divalent cations Ca^2+^ and magnesium (Mg^2+^) (13) Consistently, autosomal recessive variants in the *CLDN16* and *CLDN19* genes give rise to urinary wasting of these cations, resulting in a severe syndrome: Familial hypomagnesemia with hypercalciuria and nephrocalcinosis (FHHNC) (1, 14). These phenotypes are recapitulated in mutant mice, with excessive urinary Ca^2+^ and Mg^2+^ loss upon deletion of the *Cldn16* gene (15, 16) and CLDN16 being completely absent from the TJ in the TAL of *Cldn19*-deficient mice (17, 18). The latter suggests that CLDN19 is required to insert or retain CLDN16 within the junction, thereby explaining why pathogenic variants of *CLDN16* and *CLDN19* genes both result in FHHNC.

Elevated urinary Ca^2+^ increases the risk of kidney stone formation and is a common anomaly found in patients with kidney stone disease (19). CLDN14 is of high biomedical importance in this context, as non-coding single nucleotide variants within the *CLDN14* gene correlate strongly with both high urinary Ca^2+^ and kidney stones (20). Neither pathogenic loss-of-function *CLDN14* variants in patients nor *Cldn14* deficiency in mice lead to an altered Ca^2+^ balance, but instead result in nonsyndromic deafness (21, 22). This observation reflects the fact that CLDN14 protein expression is very low or absent in the kidney under normal conditions. Ca^2+^-sensing receptor activation increases *Cldn14* gene expression in the kidney only, when blood Ca^2+^ levels are elevated (23–28). Under these conditions, CLDN14 is found in a subset of TAL cells, where it colocalizes with CLDN16 (25). CLDN14 tightens the paracellular barrier when expressed in cell models, reducing the permeation of monovalent cations and Ca^2+^ (22, 24, 27). These findings support a model where elevated blood Ca^2+^ induces CLDN14 through Ca^2+^-sensing receptor activation, which in turn blocks Ca^2+^ permeation through CLDN16 and CLDN19 in the TAL and promotes Ca^2+^ excretion into urine. Moreover, the findings point to the likelihood that single-nucleotide *CLDN14* variants that correlate with high urinary Ca^2+^ and kidney stones cause a gain-of-function of *CLDN14* expression. How CLDN14 controls paracellular calcium flux is not yet understood on a molecular level.

**Fig. 1.**
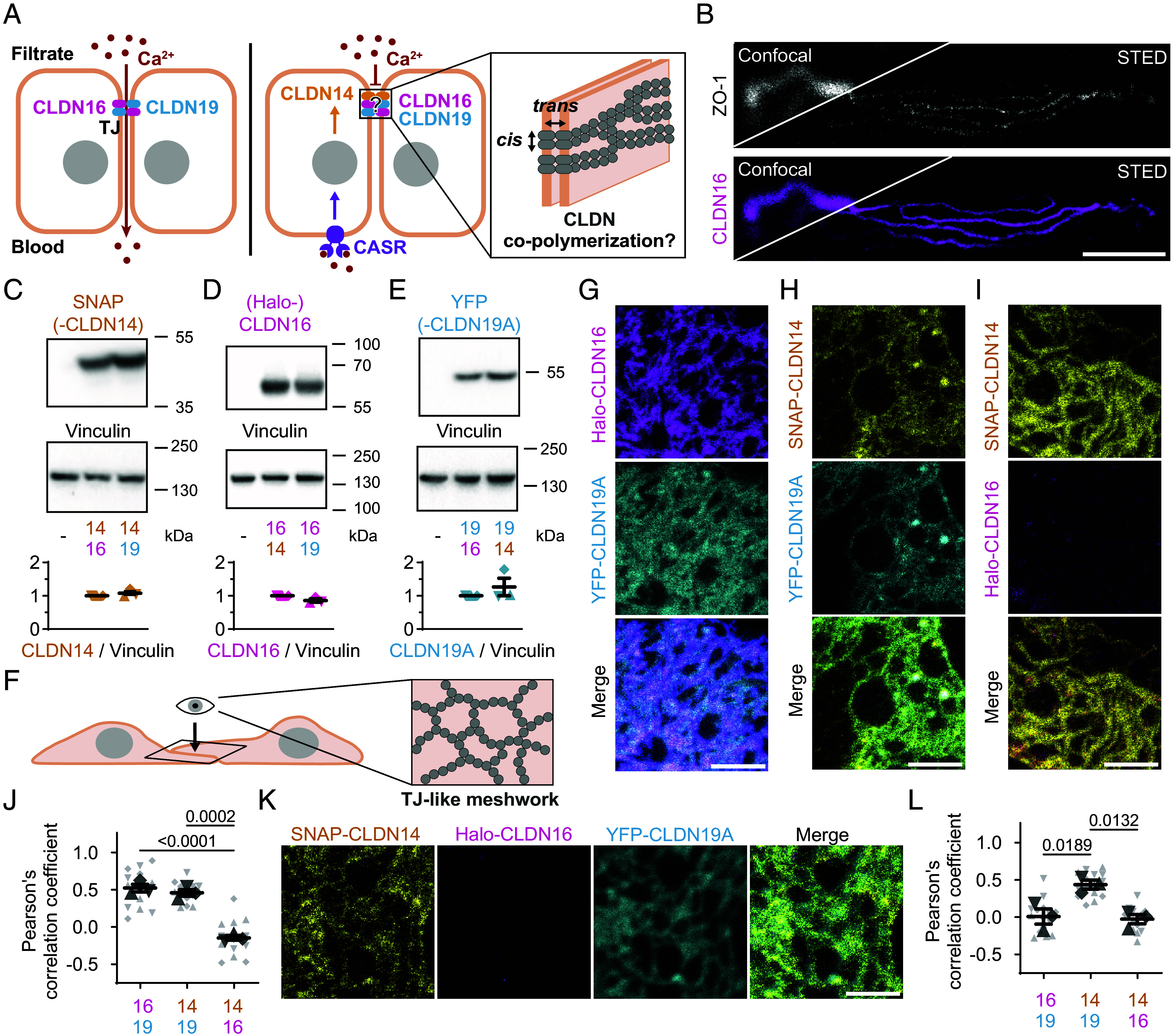
CLDN14 switches places with CLDN16 as an integral part of CLDN19 meshworks in COS-7 cells. (*A*) Model of Ca^2+^ transport through the TJ in the kidney TAL, where CLDN16 and CLDN19 copolymerize to form a Ca^2+^-permeable pore. Elevated blood Ca^2+^ activates the Ca^2+^-sensing receptor (CASR) and thereby induces CLDN14 expression, which in turn blocks the ion pore formed by CLDN16 and CLDN19. Whether and how CLDN14 copolymerizes in the TJ through interaction with claudins in the same (*cis-*interaction) or in the neighboring (*trans*-interaction) cell remains unclear. (*B*) STED-resolved TJs in the mouse kidney TAL, stained for ZO-1 (gray; Atto647N) and CLDN16 (magenta; AF594), with confocal inserts of the same region. (Scale bar: 2 µm.) (*C*–*E*) Representative immunoblots of SNAP-CLDN14 (*C*), Halo-CLDN16 (*D*), and YFP-CLDN19A (*E*) after cotransfection in COS-7 cells, with vinculin as a loading control. Claudin levels were first normalized to vinculin, then to the first cotransfection condition shown (n = 3; mean ± SEM). One-sample *t* tests: (*C*) *P* = 0.32, (*D*) *P* = 0.11, (*E*) *P* = 0.41. (*F*) Scheme demonstrating formation of a TJ-like meshwork in the overlap of two neighboring COS-7 cells exogenously expressing claudins, which can be imaged from the *top*. (*G*–*I*) STED imaging of COS-7 cells transfected with (*G*) Halo-CLDN16 (magenta; Atto590) and YFP-CLDN19A (cyan; Atto647N), (*H*) SNAP-CLDN14 (yellow; Atto590) and YFP-CLDN19A (cyan; Atto647N), and (*I*) SNAP-CLDN14 (yellow; Atto590) and Halo-CLDN16 (magenta; JF646). (Scale bars: 1 µm.) (*J*) Pearson’s correlation coefficients as a measure of colocalization of CLDN14, CLDN16, and CLDN19A in TJ-like meshworks in COS-7 cells, upon cotransfection of two claudins. Replicates (n = 3; 4 to 5 meshworks each) are represented by different symbol shapes. Mean ± SEM; one-way ANOVA (*P* < 0.0001), followed by Tukey’s multiple comparisons test; *P*-values ≤ 0.05 shown. (*K*) STED imaging of COS-7 cells transfected with SNAP-CLDN14 (yellow; Atto590), Halo-CLDN16 (magenta; JF646), YFP-CLDN19A (cyan; Atto542). (Scale bar: 1 µm.) (*L*) Pearson’s correlation coefficients as a measure of colocalization of CLDN14, CLDN16, and CLDN19A in TJ-like meshworks in COS-7 cells, upon cotransfection of all three claudins Replicates (n = 3; five meshworks each) are represented by different symbol shapes. Means ± SEM; one-way ANOVA (*P* = 0.0098), followed by a Tukey’s multiple comparisons test; *P*-values ≤ 0.05 shown.

Claudins can polymerize into intricate meshworks by interacting with other claudins in the same cell (*cis*-interaction) and in neighboring cells (*trans*-interaction) (29–31). Meshwork formation not only occurs in epithelial cells, but can also be recapitulated upon claudin overexpression in nonepithelial cells (2). Until recently, freeze-fracture electron microscopy has been the principal method for visualizing TJ meshworks (32) and revealed that the TJ in the TAL is a complex structure made up of numerous densely packed, parallel strands (33). Biochemical and cell-based studies have suggested that homomeric CLDN16 *cis*- and *trans*-interactions are either weak or nonexistent (11, 34), and that CLDN16 alone is unable to form polymeric structures in various (non)epithelial cells (13, 34–36). In the presence of CLDN19, however, an ion-permeable copolymer is created by integration of CLDN16 into CLDN19 strands (13, 34, 35). How CLDN14 interacts with or integrates into this copolymer to confer barrier properties is not known (Fig. 1*A*).

Here, we apply super-resolution stimulated emission depletion (STED) microscopy to reveal the dynamic regulation of claudin function in the complex multistranded TJ of the TAL. We use multiple in vitro and in vivo models to uncover a TJ regulatory mechanism in which CLDN14 switches places with CLDN16 within the CLDN19 copolymer, explaining how CLDN14 restricts paracellular calcium flux. Specifically, we find that CLDN19 copolymerizes with both CLDN14 and CLDN16, but preferentially associates with CLDN14 in TJ meshworks. Moreover, in a coculture system where different claudins can interact exclusively in *trans*, CLDN19 forms meshworks with CLDN14, but not CLDN16. When CLDN16 copolymerizes with CLDN19, CLDN14-dependent replacement of CLDN16 is slow and spans several days. This process exclusively occurs when CLDN14 is polymerized. Moreover, dynamin-based endocytosis is dispensable for the claudin switch. Our work offers an in-depth analysis of the regulation of a complex TJ as the one found in the TAL, and provides fundamental molecular insight into the modulation of permeability across an epithelium through the interplay of multiple claudins.

## Results

### CLDN14 Takes the Place of CLDN16 in CLDN16-CLDN19 Copolymers.

We analyzed the nanoscale molecular organization of TJs of the TAL in the mouse kidney by applying super-resolution STED microscopy with a resolution of ~50 nm. We found CLDN16 and zonula occludens-1 (ZO-1) to be organized in supramolecular structures that measured 85 ± 8 nm (mean ± SD from five STED images) in width, likely similar to the dense bundles of TJ strands previously observed with freeze-fracture electron microscopy in the rabbit TAL (33) (Fig. 1*B*). To study CLDN14, CLDN16, and CLDN19 and their copolymerization properties in detail, we coexpressed SNAP-CLDN14, Halo-CLDN16, and YFP-CLDN19A as pairs in COS-7 cells, a nonpolarized, fibroblast-like monkey cell line devoid of endogenous TJs. Each claudin was robustly expressed, independent of the specific claudin with which it was coexpressed (Fig. 1 *C*–*E*). Previous STED-based investigation of TJ-like meshworks in overlapping COS-7 cell–cell contacts (Fig. 1*F*) has revealed that CLDN14 and CLDN19 are able to homopolymerize, whereas CLDN16 required CLDN19 for polymerization (35). Applying the same approach, we reproduced the copolymerization of CLDN16 and CLDN19 (Fig. 1*G*), further demonstrated copolymerization of CLDN14 and CLDN19 (Fig. 1*H*), but observed no copolymerization of CLDN16 with CLDN14 (Fig. 1*I*). These copolymerization properties were also clearly reflected in colocalization measurements within the meshworks, showing positive correlations for CLDN16-CLDN19 and CLDN14-CLDN19, but no correlation between CLDN14 and CLDN16 (Fig. 1*J*). Strikingly, when all three claudins were coexpressed, CLDN16 no longer copolymerized with CLDN19 and was completely replaced by CLDN14 (Fig. 1*K*). As a result, only CLDN14 and CLDN19 colocalized in this condition (Fig. 1*L*). The absence of CLDN16 from the meshworks did not stem from a general loss of protein expression, as all three claudins were detected in triple-transfected COS-7 cells using both immunoblotting (*SI Appendix*, Fig. S1 *A–C*) and confocal imaging (*SI Appendix*, Fig. S1*D*). Hence, these data show that CLDN14 can replace CLDN16 within CLDN19 copolymers in COS-7 cells.

### CLDN14 alone Creates a TJ Barrier That Restricts Both Cations and Anions.

**Fig. 2.**
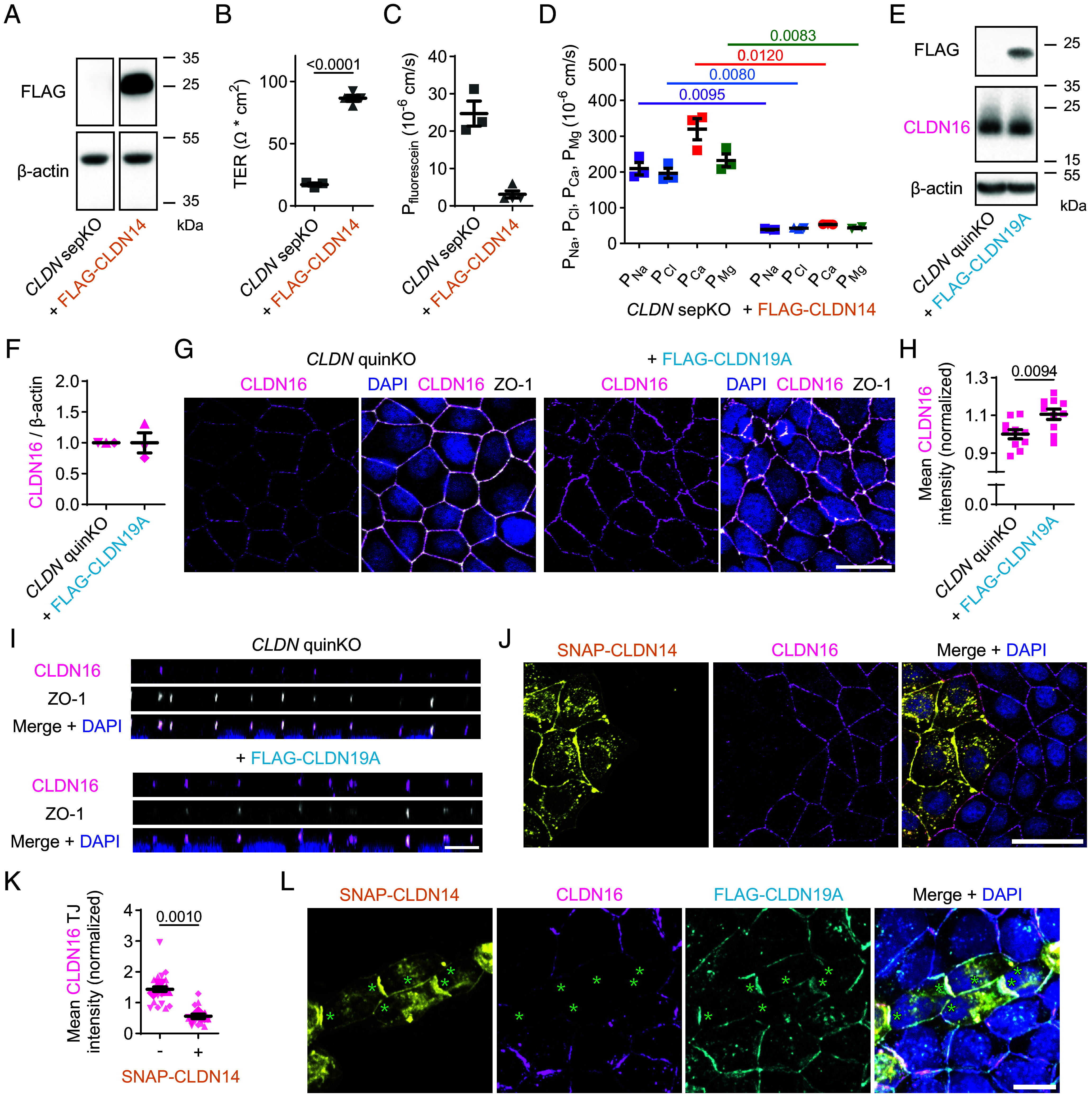
In epithelial cells, CLDN14 acts as a barrier-forming protein and can take the place of CLDN16 in the membrane. (*A*) Immunoblot to validate stable FLAG-CLDN14 expression in *CLDN* sepKO cells (that lack *CLDN1-4, CLDN7, CLDN12,* and *CLDN16*), with β-actin as a loading control. (*B*) TERs of *CLDN* sepKO monolayers with and without stable FLAG-CLDN14 expression. n = 3 to 4 filters. FLAG-CLDN14 data represent mixed cell populations obtained from two independent infection rounds, shown as upward and downward triangles. Mean ± SEM; Student’s *t* test. (*C*) Paracellular fluorescein permeability of (FLAG-CLDN14-expressing) *CLDN* sepKO monolayers. n = 3 to 4 filters and two independent infection rounds, shown with upward and downward triangles. Mean ± SEM; Mann–Whitney test (*P* = 0.0571). (*D*) Paracellular permeability of (FLAG-CLDN14-expressing) *CLDN* sepKO monolayers for Na^+^, Cl^-^, Ca^2+^, and Mg^2+^. n = 2 to 3 filters and two independent infection rounds, shown with upward and downward triangles. Mean ± SEM; Welch’s *t* tests for P_Na_, P_Cl_, P_Ca_, and P_Mg_. (*E*) Representative immunoblot showing stable FLAG-CLDN19A expression in *CLDN* quinKO cells (that lack *CLDN1-4* and *CLDN7*), as well as the endogenous levels of CLDN16 and β-actin (loading control). The same blot was probed for CLDN16, then reblotted for FLAG-CLDN19A. (*F*) Endogenous levels of CLDN16 in *CLDN* quinKO cells with and without stable FLAG-CLDN19A expression (first normalized to β-actin, then to the level in *CLDN* quinKO cells). n = 3; mean ± SEM; one-sample *t* test for the FLAG-CLDN19A expressing cells: *P* = 0.9915. (*G*) Membrane localized CLDN16 signal (magenta; AF488) in *CLDN* quinKO cells with and without stable FLAG-CLDN19A expression, costained for ZO-1 (gray; AF647) and DAPI (blue). Original z-stack: 7 images, 1 µm spacing; (Scale bar: 20 µm.) (*H*) Mean endogenous CLDN16 intensity in *CLDN* quinKO cells with or without FLAG-CLDN19A. Data were measured in maximum intensity projections and normalized to average signal in *CLDN* quinKO cells. n = 10 images; mean ± SEM; Student’s *t* test. (*I*) Side view of (FLAG-CLDN19A-expressing) *CLDN* quinKO cells, demonstrating endogenous CLDN16 (magenta; AF488) in the TJ, marked by ZO-1 (gray; AF647). DAPI (blue); original z-stacks: seven images, 1 µm spacing. (Scale bar: 10 µm.) (*J*) Endogenous CLDN16 (magenta; CF568) disappears from the membrane of *CLDN* quinKO cells upon transfection with SNAP-CLDN14 (yellow; JF646). DAPI (blue); original z-stack: eight images, 1 µm spacing; (Scale bar: 40 µm.) (*K*) Mean intensity of endogenous CLDN16 in the TJ of *CLDN* quinKO cells in the absence or presence of SNAP-CLDN14. Replicates (n = 3; five images each) are represented by different symbol shapes. For each replicate, the data were normalized to the average CLDN16 TJ signal from both conditions. Mean ± SEM; Student’s *t* test. (*L*) Maximum intensity projection of stable FLAG-CLDN19A-expressing (cyan; AF647) *CLDN* quinKO cells, 1 d after transfection with SNAP-CLDN14 (yellow; JFX554), costained for endogenous CLDN16 (magenta; AF488) and DAPI (blue), demonstrating that CLDN16 displacement by CLDN14 also occurs in the presence of CLDN19 (highlighted with green asterisks). Original z-stack: 12 images, 1 µm spacing. (Scale bar: 10 µm.)

Exogenous expression of CLDN14 increases the transepithelial electrical resistance and restricts the paracellular permeability of Ca^2+^ and sodium (Na^+^) in multiple epithelial cell models (22, 24, 27). A potential caveat of these studies is that the epithelial cell models also expressed various endogenous claudins. To avoid interference from other claudins, we developed a genome-edited MDCKII cell line completely devoid of claudins. This *CLDN* septuple knockout (sepKO) cell line was generated by further knocking out *CLDN12* and *CLDN16* in a previously established *CLDN* quintuple knockout (quinKO) cell line (that lacks *CLDN1-4* and *CLDN7*) (37) (*SI Appendix*, Fig. S2 *A–F*). Deletion of *CLDN12* and *CLDN16* did not alter junctional ZO-1 levels (*SI Appendix*, Fig. S2 *G* and *H*), but it reduced the level of occludin at the junction (*SI Appendix*, Fig. S2 *I* and *J*). Occludin levels generally seem to go down upon loss of claudins from MDCKII cells, as a similar trend was observed when generating *CLDN* quinKO cells from MDCKII cells (37). Furthermore, tricellulin levels did not differ substantially between *CLDN* quinKO and sepKO cells; however, in both cell lines its localization was not restricted to the tricellular junction as seen in MDCKII cells (*SI Appendix*, Fig. S2 *K* and *L*). Stable expression of CLDN14 in these cells (Fig. 2*A* and *SI Appendix*, Fig. S3 *A* and *B*) strengthened the TJ barrier, as evident from an increased transepithelial electrical resistance (Fig. 2*B*), and a reduced paracellular permeability for fluorescein (332 Da; Fig. 2*C*), as well as for Na^+^, chloride (Cl^-^), Ca^2+^, and Mg^2+^ (Fig. 2*D*). Thus, CLDN14 alone can form a TJ barrier which not only restricts the passage of monovalent and divalent cations but also of anions.

### Endogenous Epithelial CLDN16 Is Stabilized at the TJ by CLDN19, But Replaced by CLDN14.

We then investigated how CLDN14 might confer barrier properties in an epithelium in which TJs are already populated by CLDN16 and CLDN19. For this, we utilized genome-edited MDCKII *CLDN* quinKO cells that retain endogenous expression of CLDN16, but lack TJ strands (36, 37). We stably introduced CLDN19A into these cells (Fig. 2*E* and *SI Appendix*, Fig. S3 *C* and *D*). While this left overall CLDN16 expression unaltered (Fig. 2 *E* and *F*), it increased the CLDN16 signal at the membrane (Fig. 2 *G* and *H*), in particular in ZO-1-positive regions (Fig. 2*I*), in accordance with a recent study (13). These data are consistent with the copolymerization of CLDN16 and CLDN19 in epithelial cells.

Transient expression of SNAP-CLDN14, or stable expression of FLAG-CLDN14 (*SI Appendix*, Fig. S4 *A–C*), on the other hand, led to a marked reduction in CLDN16 at the membrane of *CLDN* quinKO cells (Fig. 2 *J* and *K* and *SI Appendix*, Fig. S4 *D* and *E*). Moreover, SNAP-CLDN14 was able to replace endogenous CLDN16 from CLDN16-CLDN19 copolymers (Fig. 2*L*), similar to what was observed in COS-7 cells (Fig. 1*K*). Full displacement of CLDN16 by CLDN14 seemed restricted to junctions between neighboring cells that both expressed CLDN14, irrespective of CLDN19 expression in these cells (Fig. 2 *J* and *L*). These data show that endogenous epithelial CLDN16 is stabilized at the TJ by CLDN19, but can be replaced by CLDN14.

### CLDN19 Slows Down CLDN16 Replacement and Preferentially Associates with CLDN14.

**Fig. 3.**
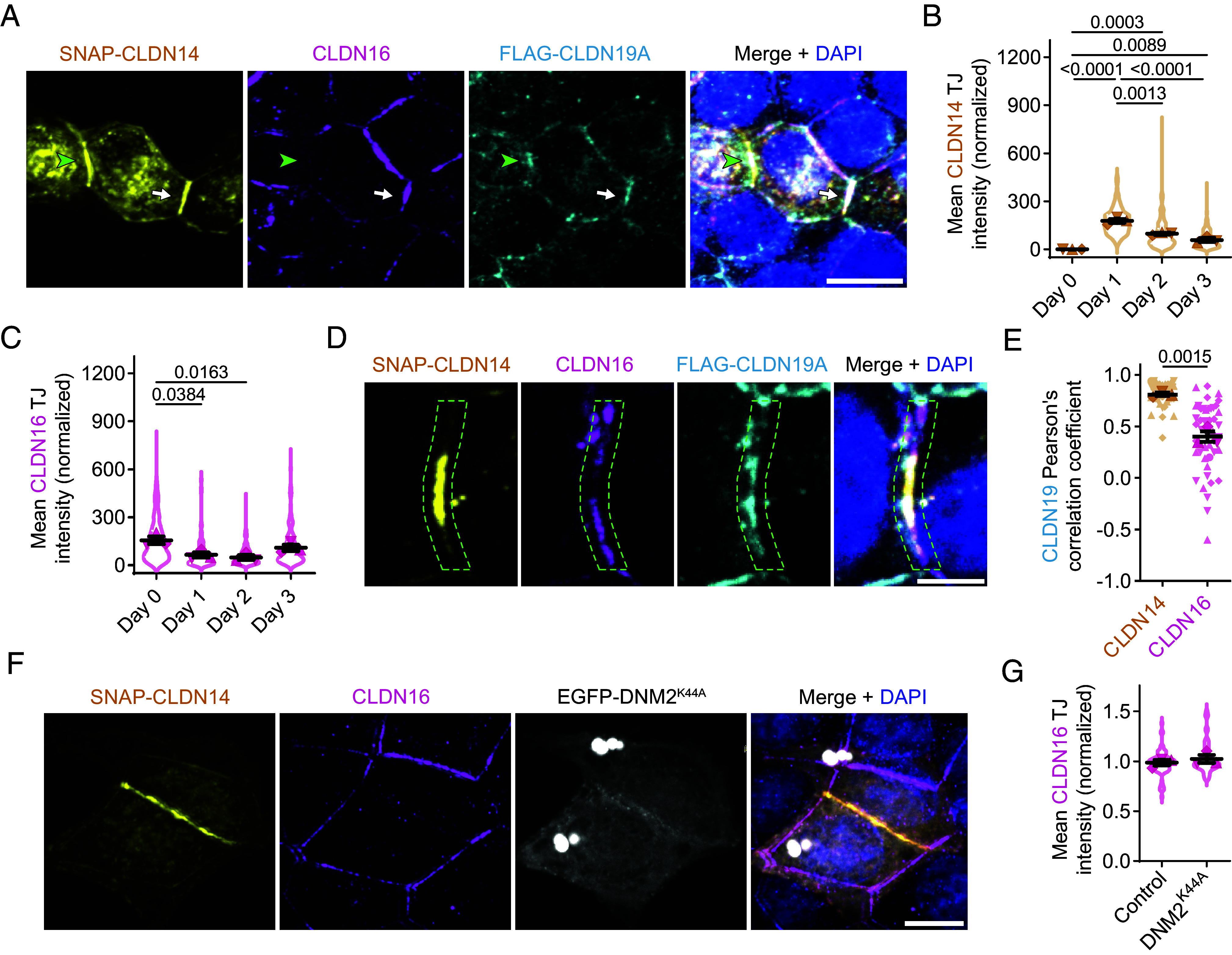
CLDN16 removal is delayed by CLDN19 copolymerization and occurs independently of dynamin-based endocytosis. (*A*) One-day SNAP-CLDN14 (yellow; JFX554) transfection in *CLDN* quinKO cells in the presence of FLAG-CLDN19a (cyan; AF647) has a variable effect on endogenous CLDN16 (magenta; AF488), sometimes fully taking its place (green arrowhead) and other times failing to do so (white arrow). DAPI (blue); original z-stack: seven images, 1 µm spacing; (Scale bar: 10 µm.) (*B* and *C*) Mean CLDN14 (*B*) and CLDN16 (*C*) intensity in the TJ of stable FLAG-CLDN19A-expressing *CLDN* quinKO cells 0, 1, 2, or 3 d after transient SNAP-CLDN14 transfection. Replicates (n = 3; 41 to 95 junctions each) are illustrated by different symbol shapes; violin plots depict the intensities in individual TJs. Per replicate, the average CLDN14 intensity from days 1, 2, and 3 was set to 100. Mean ± SEM; one-way ANOVA: (*B*) *P* < 0.0001, (*C*) *P* = 0.0155, followed by a Tukey’s multiple comparisons test. *P*-values ≤ 0.05 shown. (*D*) Example of centralized SNAP-CLDN14 (yellow; JFX554) signal 3 d after its transfection in *CLDN* quinKO cells. Immunostaining reveals that FLAG-CLDN19A (cyan; AF647) shows more overlap with the CLDN14 signal than CLDN16 (magenta; AF488) does. DAPI (blue); original z-stack: nine images, 1 µm spacing; (Scale bar: 5 µm.) (*E*) Pearson’s correlation coefficients as a measure of FLAG-CLDN19A colocalization with CLDN14 and CLDN16 in *CLDN* quinKO cell TJs that show centralized SNAP-CLDN14 signal 3 d after its transfection. Replicates (n = 3; 15 TJs each) are depicted by different symbol shapes. Mean ± SEM; Student’s *t* test. (*F*) Cotransfection of the dominant-negative endocytic mutant EGFP-DNM2^K44A^ (gray) with SNAP-CLDN14 (yellow; JFX554) does not rescue replacement of endogenous CLDN16 (magenta; AF647) in *CLDN* quinKO cells. DAPI (blue); original z-stack: seven images, 1 µm spacing. (Scale bar: 10 µm.) (*G*) Mean CLDN16 intensity in TJs between *CLDN* quinKO cells cotransfected with SNAP-CLDN14 and EGFP (control) or EGFP-DNM2^K44A^. Replicate averages (n = 3; 19 to 28 TJs each) shown with different symbol shapes; individual TJ values are depicted as a violin plot. For each replicate, values were normalized to the average CLDN16 level from the entire replicate. Mean ± SEM; Student’s *t* test: *P* = 0.4833.

Comparing *CLDN* quinKO cells with and without FLAG-CLDN19A, we noticed that the replacement of CLDN16 by SNAP-CLDN14 happened at different rates in the two cell types. When CLDN19 was absent, a complete switch between CLDN16 and CLDN14 was consistently observed 1 d posttransfection (Fig. 2*J*). In contrast, when CLDN19 was present, some junctions showed complete replacement at this timepoint, while others did not, with the degree of replacement depending on the level of CLDN14 expression (Fig. 3*A*). To dissect the temporal and spatial evolution of claudin switching in stable FLAG-CLDN19A-expressing *CLDN* quinKO cells, we quantified TJ levels of CLDN14 and endogenous CLDN16 over a 3-d period following transient expression of SNAP-CLDN14. As expected, CLDN14 levels peaked 1 d posttransfection and then gradually decreased (Fig. 3*B*). Junctional CLDN16 levels were decreased 1 d posttransfection, but were lowest 2 d posttransfection, with recovery occurring only by day three (Fig. 3*C*). The altered turnover kinetics of CLDN16 in *CLDN* quinKO cells, depending on FLAG-CLDN19A expression, imply that copolymerization with CLDN19 slows down CLDN16 replacement. During TJ repopulation by CLDN16 3 d posttransfection, CLDN16 appeared from the periphery of the TJ, while CLDN14 localized more centrally (Fig. 3*D*). In these junctions containing all three claudins, CLDN19 preferentially colocalized with CLDN14 rather than with CLDN16 (Fig. 3*E*). These results suggest that claudin switching may occur by a competitive mechanism in which CLDN19 binds with preference to CLDN14 over CLDN16.

### The Switch from CLDN14 to CLDN16 Is Independent of Dynamin-Mediated Endocytosis.

One possible way by which CLDN14 may trigger CLDN16 TJ removal and replacement is by inducing its endocytosis and subsequent degradation. Clathrin- and dynamin-mediated endocytosis has been previously identified as a key pathway for the internalization of CLDN16 in MDCKII cells (38). We used a dominant-negative approach to interfere with this endocytic pathway based on the expression of a GTP hydrolysis-defective mutant of dynamin-2 (DNM2^K44A^), a key factor for the scission of endocytic vesicles. Although DNM2^K44A^ successfully impaired endocytosis in *CLDN* quinKO cells, as evidenced by marked suppression of transferrin uptake (*SI Appendix*, Fig. S5 *A* and *B*), it did not prevent CLDN16 replacement by CLDN14 in our cell model (Fig. 3 *F* and *G*). Collectively, this demonstrates that CLDN16 replacement is independent of dynamin-mediated endocytosis.

### CLDN14 Copolymerization with CLDN19 Plays a Crucial Part in the Claudin Switch.

**Fig. 4.**
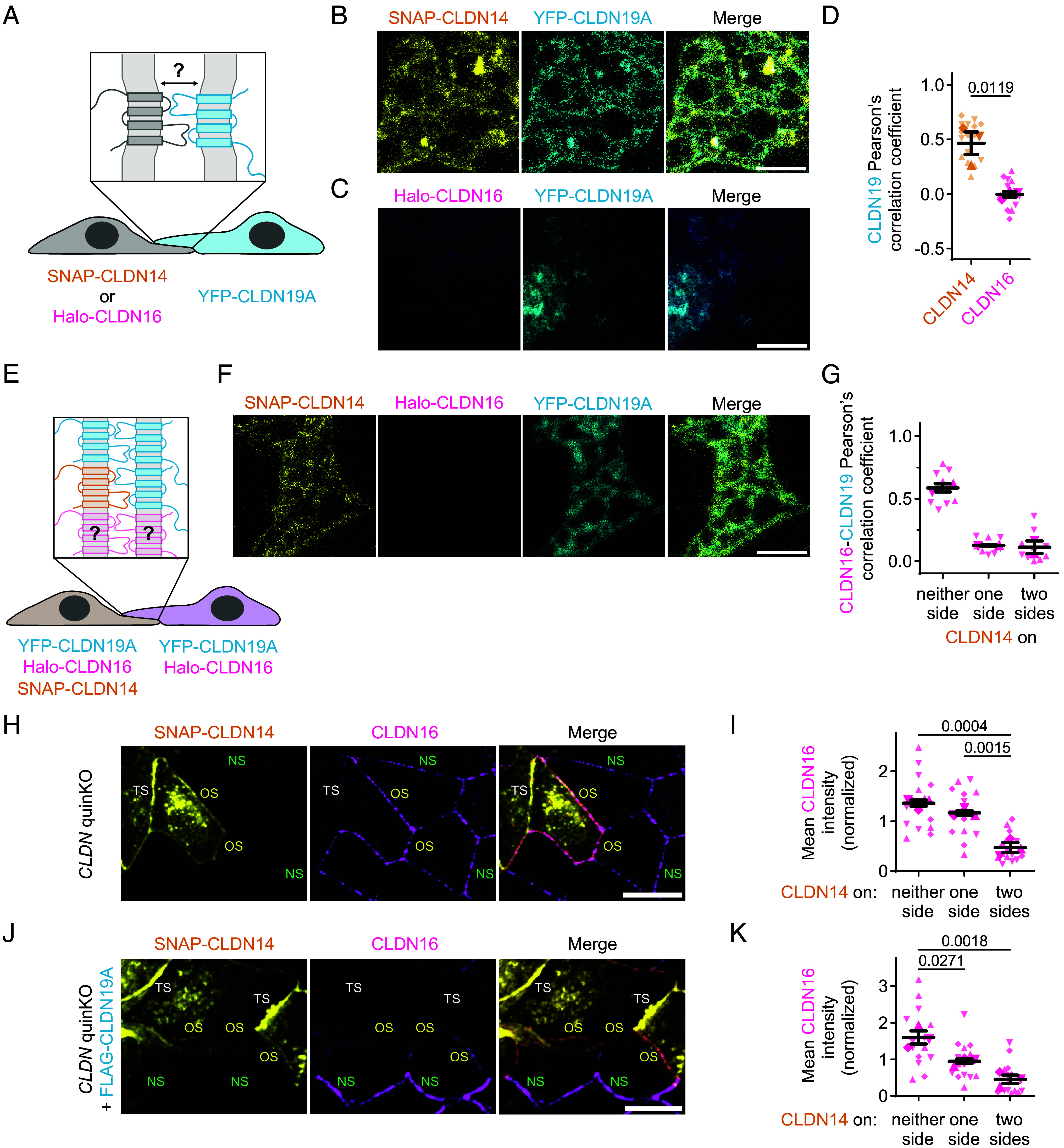
Claudin (co)polymerization properties and their role in the claudin switch. (*A*) Schematic representation of the COS-7 coculture system designed to evaluate *trans*-interaction-dependent meshwork formation between cells expressing SNAP-CLDN14 or Halo-CLDN16 and those expressing YFP-CLDN19A. (*B* and *C*) STED images of cell–cell overlaps between COS-7 cells transfected with (*B*) SNAP-CLDN14 (yellow; Atto590) or YFP-CLDN19A (cyan; Atto647N), and (*C*) Halo-CLDN16 (magenta; Atto590) or YFP-CLDN19A (cyan; Atto647N). (Scale bars: 1 µm.) Confocal overview images of the two cells forming each cell–cell overlap shown in *SI Appendix*, Fig. S6 *A* and *B*. (*D*) Pearson’s correlation coefficients as a measure of YFP-CLDN19A colocalization with either SNAP-CLDN14 or Halo-CLDN16 in overlaps between cocultured COS-7 cells. Replicates (n = 3; 4 to 5 meshworks each) are represented by different symbol shapes. Means ± SEM; Student’s *t* test. (*E*) Schematic representation of the COS-7 coculture system to evaluate whether CLDN14 incorporation from only one side of the CLDN16-CLDN19A meshwork is sufficient to remove CLDN16. (*F*) STED image of a cell–cell overlap between two COS-7 cells which are both transfected with Halo-CLDN16 (magenta; JF646) and YFP-CLDN19A (cyan; Atto542), but only one is transfected with SNAP-CLDN14 (yellow; Atto590; balanced by pcDNA3.1(+) in the other). (Scale bar: 1 µm.) A confocal overview image of the two cells is shown in *SI Appendix*, Fig. S7*E*. (*G*) Pearson’s correlation coefficients as a measure of Halo-CLDN16 and YFP-CLDN19A colocalization in TJ-like meshworks in COS-7 cells, with coexpression of SNAP-CLDN14 on neither, one or two of the sides of the meshwork. Replicates (n = 2; 3 to 5 meshworks each) represented by different symbol shapes; mean ± SEM. (*H*–*K*) Endogenous CLDN16 signal in *CLDN* quinKO cells (*H* and *I*) and FLAG-CLDN19A–expressing *CLDN* quinKO cells (*J* and *K*) with SNAP-CLDN14 expressed on two sides (TS), one side (OS), or neither side (NS) of the junction. (*H* and *J*) Maximum intensity projections show that in *CLDN* quinKO cells [*H*; eight images, 1 µm spacing; (Scale bar: 20 µm)] CLDN16 (magenta; CF568) is reduced only with two-sided (TS) CLDN14 (yellow; JF646) expression, whereas in FLAG-CLDN19A–expressing *CLDN* quinKO cells [*J*; 10 images, 1 µm spacing; (Scale bar: 10 µm); FLAG-CLDN19A shown in *SI Appendix*, Fig. S6*C*)] CLDN16 (magenta; AF488) is fully lost in junctions with CLDN14 (yellow; JFX554) on both sides (TS), but also reduced in junctions with one-sided CLDN14 (OS). (*I* and *K*) Mean CLDN16 intensity in *CLDN* quinKO (*I*) and FLAG-CLDN19A–expressing *CLDN* quinKO (*K*) cells that express CLDN14 on neither side (NS), one side (OS), or two sides (TS) of the junction. Replicates (n = 3; five images each) represented with different symbol shapes; mean ± SEM; one-way ANOVA: (*I*) *P* = 0.0004, (*K*) *P* = 0.0022, followed by Tukey’s multiple comparisons tests; *P* ≤ 0.05 shown.

Rather than actively targeting CLDN16 for endocytic removal from the TJ, CLDN14 may outcompete it through preferential copolymerization with CLDN19. Claudin polymerization relies on both *cis*- and *trans*-interactions (Fig. 1*A*) (29–31). Our original STED imaging of copolymerization in COS-7 cells showed that both CLDN14 and CLDN16 can copolymerize individually with CLDN19 (Fig. 1 *G* and *H*). However, it did not resolve the distinct roles of *cis*- and *trans*-cellular interactions. To address the role of *trans*-interactions, we employed a COS-7 coculture system with CLDN19 expressed in one cell population and CLDN14 or CLDN16 in the other (Fig. 4*A*). Strikingly, CLDN14 and CLDN19 formed meshworks through a heterotypic *trans*-interaction, while no such structures were seen between CLDN16- and CLDN19-expressing COS-7 cells (Fig. 4 *B*–*D* and *SI Appendix,* Fig. S6 *A–C*); the latter matching the previously reported absence of *trans*-interaction in HEK293 cells (11). This means that, at least in *trans*-interactions with CLDN19, CLDN14 holds a distinct advantage over CLDN16.

Should CLDN14 replace CLDN16 because it (co)polymerizes easier, incorporation of CLDN14 on only one side of the junction may suffice to displace CLDN16. We tested this in a COS-7 coculture system where all cells expressed CLDN16 and CLDN19, but only half of them expressed CLDN14 (Fig. 4*E*). In this coculture, we could reproduce the copolymerization of CLDN16 and CLDN19 in the absence of CLDN14 (*SI Appendix*, Fig. S7 *A* and *B*), and the disappearance of CLDN16 from the meshwork when both cells expressed CLDN14 (*SI Appendix*, Fig. S7 *C* and *D*). In agreement with the polymerization strength rationale, CLDN16 was completely absent from meshworks formed between COS-7 cells of which only one expressed CLDN14 (Fig. 4*F* and *SI Appendix*, Fig. S7*E*), leading to comparable outcomes for CLDN16-CLDN19 colocalization whether CLDN14 was present on one or on both sides (Fig. 4*G*). Thus, at least in COS-7 cells, CLDN14 can drive CLDN16 removal from copolymers both in the cell where they are coexpressed and in the adjoining cell without CLDN14.

Finally, underscoring the role of CLDN14 (co)polymerization in CLDN16 removal, we noted distinct CLDN16 replacement patterns in *CLDN* quinKO cells depending on the presence or absence of CLDN19. Specifically, replacement only occurred at junctions where CLDN14 could establish *trans*-interactions required for polymerization. In the absence of other (co)polymerizing claudins, CLDN14 in *CLDN* quinKO cells (without CLDN19) can polymerize solely with itself (as seen in COS-7 cells in Fig. 1*I*); i.e., CLDN14 polymers will form in junctions between two CLDN14-expressing cells [two-sided (TS) junctions], in which CLDN14 is its own *trans*-interacting partner. In FLAG-CLDN19A-expressing *CLDN* quinKO cells, CLDN19 serves as an additional CLDN14 copolymerization partner. Because CLDN19 is expressed in all cells, CLDN14 can form polymers in junctions with CLDN14 on both sides (TS junctions), or just on one side (OS junctions), where CLDN19 copolymerizes in *trans* [as seen in COS-7 cells (Fig. 4*B*)]. We found that in *CLDN* quinKO cells, CLDN16 removal occurred only at two-sided (TS) junctions (Fig. 4 *H* and *I*), whereas in FLAG-CLDN19A-expressing cells it was also removed from one-sided (OS) junctions (Fig. 4 *J* and *K* and *SI Appendix*, Fig. S7*F*), precisely matching CLDN14 polymerization sites. In contrast to COS-7 cells, where CLDN16 is fully replaced with CLDN14 on only one side of the meshwork (Fig. 4*F*), FLAG-CLDN19A-expressing cells showed incomplete removal of CLDN16 from one-sided (OS) junctions (Fig. 4*J*). This discrepancy presumably stems from the fact that in epithelial cells, unlike in COS-7 cells, additional regulatory processes like signaling and trafficking are involved in delivering proteins to the TJ meshwork. Overall, these data point to a central role for claudin (co)polymerization in the transition from CLDN16 to CLDN14.

### Upon Triggering CLDN14 Expression in the Mouse TAL, the Claudin Switch Is Evident In Vivo.

**Fig. 5.**
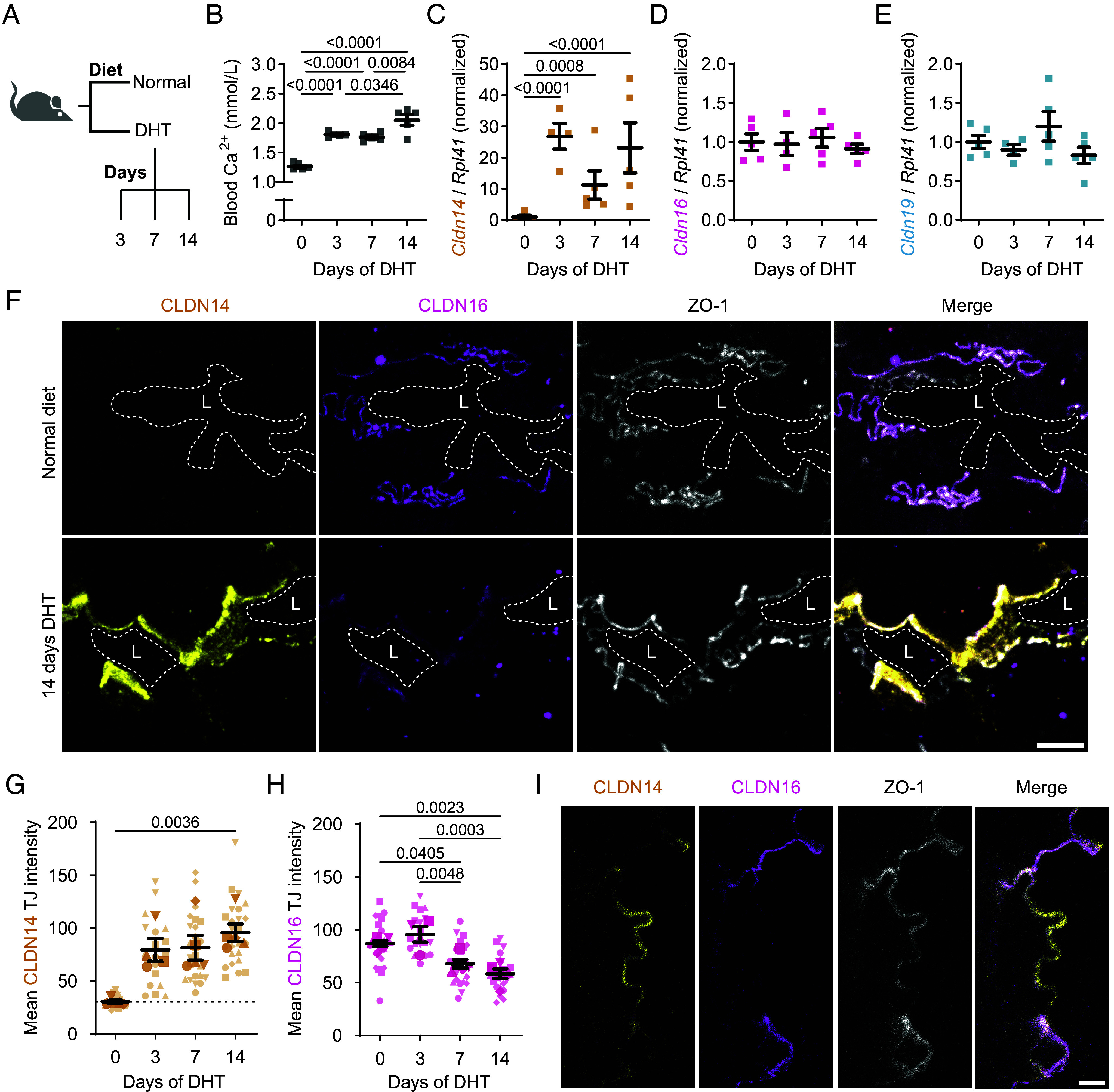
Confocal and STED microscopy reveal CLDN16 replacement by CLDN14 in the mouse TAL in vivo. (*A*) Scheme of experimental setup with mice on a normal diet or on a DHT diet for 3, 7, or 14 d. (*B*) Blood Ca^2+^ levels in mice after different durations of the DHT diet. n = 4 to 5; mean ± SEM; one-way ANOVA (*P* < 0.0001), followed by a Tukey’s multiple comparisons test; *P*-values ≤ 0.05 shown. (*C*–*E*) Renal *Cldn14* (*C*), *Cldn16* (*D*), and *Cldn19* (*E*) mRNA expression levels in mice subjected to increasing durations of a DHT diet (first normalized to the mRNA expression levels of *Rpl41*, then to the average levels in mice without DHT). n = 4 to 5; means ± SEM; one-way ANOVA: (*C*) *P* < 0.0001, (*D*) *P* = 0.8172, (*E*) *P* = 0.3292, followed by Tukey’s multiple comparisons tests; *P*-values ≤ 0.05 shown. (*F*) Example maximum intensity projections of the TAL from a mouse on a normal diet versus one on a 14-d DHT diet, with TJs lining the lumen (L) marked by ZO-1 (gray; Atto647N). The DHT diet induces CLDN14 expression (AF488; yellow) and appears to reduce CLDN16 levels (magenta; AF594). Original z-stack: six images, 1 µm spacing; (Scale bar: 5 µm.) (*G* and *H*) Mean CLDN14 (*G*) and CLDN16 (*H*) intensity in TAL TJs of mice subjected to increasing durations of a DHT diet. Different symbol styles correspond to different mice (n = 4 to 5; five TALs analyzed each). Mean ± SEM; statistical tests: (*G*) Kruskal–Wallis test (*P* ≈ 0.0065) followed by a Dunn’s multiple comparisons test, (*H*) one-way ANOVA (*P* = 0.0002) followed by a Tukey’s multiple comparisons test. *P*-values ≤ 0.05 shown. The dotted line in (*G*) indicates the absence of CLDN14. (*I*) STED image of the TJ region adjacent to the TAL lumen in a 14-d DHT fed mouse, stained for CLDN14 (yellow; AF488), CLDN16 (magenta; AF594), and ZO-1 (gray; Atto647N). (Scale bar: 1 µm.)

Our findings demonstrate that CLDN14 can substitute CLDN16 in COS-7 and epithelial cells in vitro. To test whether the claudin switch exists in the complex TAL tight junction in vivo, mice were fed with the vitamin D analog dihydrotachysterol (DHT) for 3, 7, or 14 d (Fig. 5*A* and *SI Appendix*, Table S1). This increased blood Ca^2+^ levels (Fig. 5*B*), resulting in increased *Cldn14* messenger RNA (mRNA) expression in the mouse kidney (Fig. 5*C*), while *Cldn16* and *Cldn19* mRNA levels remained unchanged (Fig. 5 *D* and *E*). More prominent CLDN14 levels were detected in the TAL in kidney sections of mice fed with DHT, mostly colocalized with the TJ marker ZO-1 (Fig. 5*F*). Additionally, CLDN14 localized to apical membrane domains, as it did upon overexpression in *CLDN* sepKO or quinKO cells (*SI Appendix*, Figs. S3*B* and S4*C*). In agreement with our in vitro data, CLDN14 elevation was paralleled by a decrease of CLDN16 in the TJ (Fig. 5*F*). Quantification of replacement dynamics revealed that while CLDN14 TJ levels increased after 3 d of DHT treatment (Fig. 5*G*), CLDN16 downregulation required prolonged DHT exposure (7 or 14 d) (Fig. 5*H*). Reversal of the replacement also seems to take a considerable amount of time. Following a 14-d DHT diet, a 4-d normal diet was sufficient to nearly return blood Ca^2+^ levels to baseline (*SI Appendix*, Fig. S8 *A* and *B*). However, this period was too short to observe a prominent reduction in junctional CLDN14 (*SI Appendix*, Fig. S8*C*) or recovery of CLDN16 (*SI Appendix*, Fig. S8*D*), in line with a slow, dynamic replacement in vivo. Finally, super-resolution STED microscopy on the TAL from 14-d DHT-treated mice occasionally revealed distinct TJ domains populated by CLDN14 and CLDN16 (Fig. 5*I*). Overall, these data recapitulate the gradual displacement of CLDN16 at CLDN14-positive junctions, as observed in our in vitro models, and identify a mechanism by which increased CLDN14 expression alters the permeability properties of the TAL.

## Discussion

CLDN14 in the TAL increases urinary Ca^2+^ excretion to protect against excess Ca^2+^ in the blood (24, 25, 27). Notably, variants in the *CLDN14* gene strongly correlate with elevated urinary Ca^2+^ excretion and kidney stone disease (20), likely through upregulation of *CLDN14* expression. CLDN19 promotes stable CLDN16 incorporation into the TJ to form a Ca^2+^-permeable pore in the TAL (13, 17, 18, 34, 35), but how CLDN14 blocks this pore to promote Ca^2+^ excretion is unknown. In this study, we show that CLDN14 acts by copolymerization with CLDN19 to replace CLDN16 in the TJ. With STED microscopy, the claudin switch was observed not only in claudin polymers in COS-7 cells in vitro but also within TJ segments of the mouse TAL in vivo. Using the highest-resolution fluorescence imaging of the TAL to date, we present a dynamic mechanism controlling tight junction permeability.

Claudin expression in the TAL is mosaic with CLDN16 and CLDN19 localizing to distinct junctions (10, 11). CLDN14, when upregulated, is found in the same cell type that expresses CLDN16 (23, 25). Consequently, CLDN14 was thought to block Ca^2+^-pores through copolymerization with CLDN16 and CLDN19 at the TJ, but this hypothesis has not been experimentally validated. In contrast, our study reveals that CLDN14 replaces CLDN16. Other noteworthy aspects of this work are the exploration of heteromeric claudin channel (CLDN16-CLDN19) regulation through substitution of the pore-forming component (13) and the in vivo demonstration of this process. Previous studies have either only theoretically described claudin switching (39), or focused solely on the replacement of homomeric cation channel-forming CLDN2 in in vitro models (40–42). In addition, we identify CLDN14 as a barrier-forming claudin in a claudin-free epithelial cell model. Thus, claudin switching not only removes the pore-forming component of the CLDN16-CLDN19 complex (13) but also introduces a second barrier-forming claudin alongside CLDN19 (13).

In *CLDN* quinKO cells, replacement occurred within 24 h when CLDN16 was not polymerized. In contrast, when CLDN16 was coexpressed and thus copolymerized with CLDN19, it took several days. This process was even slower in vivo, where a 7- or 14-d DHT diet was required to observe significant CLDN16 removal from the TJ. In contrast, a 3-d diet, used to increase CLDN14 expression in the past (25, 28), was sufficient to detect CLDN14 in the TJ but too short to detect significant changes in junctional CLDN16. The delay could theoretically stem from slow gene regulatory mechanisms, but this seems unlikely, as overall *Cldn16* expression did not change over the course of the DHT diet. The TJ in the TAL consists of densely packed parallel strands (33). In cellular models analyzed by freeze-fracture electron microscopy, CLDN19 expression recreates the parallel strand organization (24, 43), with the addition of CLDN16 reducing the space between the strands (24). In *CLDN* quinKO cells specifically, stable expressed CLDN19 creates a large meshwork that extends laterally (13). Studies addressing the turnover of TJs of such architectural complexity have yet to be conducted. By comparison, a study of the much simpler TJs of MDCKII cells, characterized by loose meshes and few parallel strands (44), has shown complete turnover after 24 h (45). The complex TJ structure in the TAL may thus require several days to turn over fully, with initial partial CLDN16 removal being too subtle to detect.

In CLDN14-, and CLDN16-containing TJs in *CLDN* quinKO cells, CLDN19 preferentially associates with CLDN14. This contrasts with prior research, which claimed CLDN14 and CLDN19 only interact indirectly through CLDN16 (24). That study, however, was limited to examining *cis*-interactions in unpolarized yeast and HEK293 cells. As we demonstrate in this study, CLDN14 has a distinct advantage over CLDN16 in forming *trans*-interactions with CLDN19. Our experiments take into account all interactions that occur during TJ polymer assembly, including such *trans*-interactions. Moreover, many of our experiments are conducted in epithelial cells, introducing an extra layer of regulation via polarized protein trafficking. Thus, we believe our data better reflect physiologically relevant claudin–claudin interaction patterns.

Perturbing dynamin, a central component of clathrin-mediated endocytosis, did not prevent CLDN16 removal in our experiments, despite previous evidence showing that MDCK cells internalize CLDN16 via clathrin-coated pits (38). Although removal via other endocytic pathways cannot be ruled out, the fact that only polymerized CLDN14 triggers replacement implies regulation at the stages of TJ polymerization and/or membrane targeting. CLDN16 depends on CLDN19 for incorporation in the TJ (13, 17, 18, 34, 35). In our experiments, CLDN19 nevertheless favors interaction with CLDN14, which may leave CLDN16 stranded in the membrane or vesicular compartments. Alternatively, CLDN14 may outcompete CLDN16 for binding to ZO-1, a cytosolic TJ adapter. ZO-1 association is essential for TJ localization of various claudins (37, 46). This includes CLDN16, as one of the FHHNC-causing variants in *CLDN16* disrupts ZO-1 binding, resulting in lysosomal CLDN16 accumulation (47). Future work should focus on a deeper mechanistic understanding of the claudin switch in the TAL.

While our study centered on blood Ca^2+^-dependent regulation of CLDN14 expression through Ca^2+^-sensing receptor activation, parathyroid hormone also modulates CLDN14 in the TAL. In mice lacking the parathyroid hormone 1 receptor in the distal tubule, hypercalciuria develops alongside increased *Cldn14* expression and this phenotype is rescued upon *Cldn14* deletion (48). Determining the interplay between these regulatory mechanisms should be clarified in the future.

As the levels of transiently transfected CLDN14 in *CLDN* quinKO cells declined after a few days, CLDN16 returned to the TJ, demonstrating that the claudin switch is reversible in epithelial cells. In agreement with this, urinary Ca^2+^ excretion in vivo is believed to be dynamically regulated by the Ca^2+^-sensing receptor, ensuring stable blood Ca^2+^ levels. However, in mice, a 4-d normal diet following a 14-d DHT diet was nearly sufficient to normalize blood Ca^2+^ levels, but insufficient to reduce CLDN14 and increase CLDN16 levels at the junction. We speculate that CLDN14 expression may already be reduced at this point due to rapid gene regulation, yet junctional levels remain unaffected because the junctions turn over slowly. Given that CLDN16 took 7 d to be removed from the junction, CLDN14 may follow a comparable timescale. To investigate whether and how CLDN16 can return to the TJ in vivo, future studies should be conducted over a prolonged period of time.

During TJ repopulation by CLDN16 in epithelial cells, CLDN16 was most prominent on the sides of the meshwork, and CLDN14 accumulated centrally. This could arise from differences in turnover across the meshwork, driven by factors like higher tension at tricellular tight junctions due to the convergence of three cells [as reported for tricellular adherens junctions (49)], or more active remodeling at the edges. If CLDN16 is also concentrated in the central part of the meshwork as it disappears from the polymers, removing most of it would be necessary to limit Ca^2+^ permeation through CLDN16-CLDN19 copolymers. Downregulation of CLDN16 alone would therefore not efficiently reduce paracellular Ca^2+^ flux, explaining the necessity of separate upregulation of barrier-forming CLDN14. The integration of CLDN14 in as few as one or two strands along the full width of the TJ could swiftly block Ca^2+^ flux. Moreover, in vitro CLDN16 removal happens even in TJs where CLDN14 is present on just one side, since CLDN19 in the opposing cells enables CLDN14 polymerization via *trans*-interaction. Thus, CLDN14 expression in only half of the TAL cells could even suffice to block Ca^2+^ flux.

Based on the existence of basolateral claudin pools in various tissues (50) and live imaging of claudin turnover in MDCKII cells (45), a model has been proposed where claudins are added to meshworks basolaterally. It has been proposed that basolateral claudin pools serve as a signaling hub (50), and CLDN10B in particular stabilizes basolateral invaginations in the TAL to facilitate salt transport (51), though their exact roles at these compartments remain to be clarified. Contrary to this model, we observe apical CLDN14 localization upon overexpression in *CLDN* quinKO and sepKO cells and endogenously in the mouse kidney. Other studies in mice have likewise found apical CLDN14 staining in the TAL, with specificity confirmed by its loss in *Cldn14*-deficient animals (23, 25, 52). It is believed that the apical presence of monomeric or *cis*-oligomeric claudins facilitates binding of the enterotoxin of *Clostridium perfringens* (which causes food poisoning) to epithelial cells (53). However, to our knowledge, the apical localization of an endogenous claudin has otherwise only been reported once, for CLDN4 in HT-28/B6 cells (54). Reports of other claudins localizing apically exist, though always upon overexpression in cell culture (55, 56). Along the same lines, in TJs of the inner ear, CLDN14 forms a morphologically distinct domain apically from CLDN6 and CLDN9 (57). Thus, apical localization seems to be a distinctive feature of CLDN14. One possibility is that during TJ turnover, CLDN14, unlike other claudins, is primarily inserted into meshworks from the apical side. Basolateral incorporation in vitro can however, not be entirely dismissed, as a basolateral pool is detectable in our *CLDN* KO cells (but not in the mouse kidney). Dual-sided incorporation, which has been documented in the context of *Drosophila* glial septate junctions (58), could speed up the overall process of CLDN14 incorporation and subsequent CLDN16 removal. Another possibility is that CLDN14 serves a signaling function at the apical membrane, just as proposed for basolateral claudins. Properly understanding the role of CLDN14 at the apical membrane will require additional targeted investigation.

Overall, this study sheds light on the molecular regulation of epithelial permeability, revealing how a switch in claudin polymer composition shapes the characteristics of complex tight junctions. More specifically, it enhances our understanding of Ca^2+^ regulation in the TJ of the kidney TAL, introducing CLDN16 replacement by CLDN14 as a regulatory mechanism. Modulating this process holds potential for therapeutic interventions in conditions with increased urinary Ca^2+^ excretion and kidney stone disease.

## Materials and Methods

In this section, the materials and methods are summarized in a comprehensive manner. Detailed information if provided in the *SI Appendix*.

### Constructs.

Plasmids used in this study: Halo-CLDN16, YFP-CLDN19A, YFP-CLDN12, YFP-CLDN16, SNAP-CLDN14, pCIG3.NB, pMD2.G, pEGFP-N1, EGFP-Dynamin 2 K44A, pLIB-CMV-FLAG-CLDN14-Puro, and pLIB-CMV-FLAG-CLDN19A-Puro.

### Cell Culture and Cell Line Generation.

The following cell lines were used: COS-7 (ATCC CRL 1651), HEK293T (ATCC CRL 11268), MDCKII (ECACC 00062107), and MDCKII claudin quintuple knockout (courtesy of Prof. Mikio Furuse, National Institute for Physiological Sciences, Japan). Cells were cultured in high-glucose Dulbecco’s Modified Eagle Medium with 10% fetal bovine serum and 100 μg/mL penicillin-streptomycin at 37 °C in 5% CO_2_. Cells were seeded on μ-Slide 8-well glass-bottom dishes, 25 mm #1.5H precision coverslips, in 6-well plates, or on 12 mm polycarbonate Millicell inserts. Transfections were performed with Lipofectamine 2000.

MDCKII *CLDN* sepKO cells were generated from MDCKII *CLDN* quinKO cells by disrupting the *CLDN12* and *CLDN16* genes using CRISPR-Cas9. After transfection with plasmids encoding gRNAs and Cas9, cells were selected with puromycin, single-cell–derived colonies were isolated, and clones were expanded. Successful knockout of *CLDN12* and *CLDN16* was validated with immunoblotting and immunocytochemistry.

Stable cell lines were generated by retroviral transduction of MDCKII-derived cells with pLIB-CMV-FLAG-CLDN constructs. Transduced cells were selected with puromycin and maintained as mixed populations without clone picking.

### SNAP- and Halo-Tag Labeling and Transferrin Uptake.

Cells were labeled with 1 to 2 μM SNAP- or Halo-tag substrate for 30 to 60 min at 37 °C, followed by extensive washing before fixation or imaging. Transferrin-AF647 uptake assays were conducted in 2 h serum-starved cells for 30 to 60 min at 37 °C.

### Animal Experiments.

Female FVB/N mice were maintained on a standard rodent diet. Hypercalcemia was induced by supplementing the diet with DHT for defined durations. Toward the end of the experiments, mice were placed in metabolic cages for measurements. At the endpoint, mice were anesthetized with 1.5% isoflurane, blood was collected from the vena cava, and kidneys were harvested; one half was fixed in 4% paraformaldehyde for immunohistochemistry, and the other half frozen for RNA extraction.

Blood ionized Ca^2+^ concentrations were measured using an ABL835 analyzer. Urinary Ca^2+^ concentrations were determined by ion chromatography, and creatinine levels were quantified using an ABX Pentra Creatinine 120 CP kit.

### RNA Extraction and qPCR.

Total RNA was extracted from kidney tissue using TRIzol reagent and treated with DNase I to remove genomic DNA. Complimentary DNA was synthesized, and quantitative PCR was performed on a real-time PCR system. Gene expression was normalized to the housekeeping gene *Rpl41*, and relative expression levels were calculated using the 2^−ΔΔCt^ method.

### Fixation, Immunocytochemistry, and Immunohistochemistry.

COS-7 cells were fixed with 4% paraformaldehyde and 4% sucrose, MDCKII-derived cells with ice-cold ethanol. Cells were permeabilized (if needed), blocked with normal goat serum and bovine serum albumin, incubated with primary antibodies and fluorescent secondary antibodies, and nuclei were stained with DAPI.

Fixed kidneys were dehydrated, and embedded in paraffin before sectioning at 5 μm. Sections were rehydrated, antigens retrieved, and endogenous aldehydes and peroxidases blocked. Primary antibodies were incubated overnight at 4 °C, followed by secondary antibody staining. Biotinylated antibodies were detected using fluorophore-conjugated streptavidin, and sections were mounted in antifade medium.

### Cell Lysis and Immunoblotting.

Cells were lysed in Triton X-100–containing buffer, and lysates were cleared by centrifugation at 17,000 g. Protein concentrations were determined by Bradford assay. Equal amounts of protein were denatured at 95 °C in sodium dodecyl sulfat (SDS) sample buffer, separated by SDS polyacrylamide gel electrophoresis, and transferred to nitrocellulose membranes. Membranes were blocked and incubated with primary and horseradish peroxidase-conjugated secondary antibodies. Detection was performed using chemiluminescent substrate, and protein levels were quantified.

### Transepithelial Electrical Resistance, Fluorescein, and Ion Permeability Measurements.

TER and ion permeability measurements were performed in Ussing chambers. Bicarbonate-free Ringer’s solution was used throughout to prevent Ca^2+^ precipitation. TER values were first recorded. Transepithelial voltages were recorded after apical mannitol addition (for Na^+^, Cl^−^) or sequential basolateral ion application (for Ca^2+^, Mg^2+^), and permeabilities were derived as previously described (59). For fluorescein permeability measurements, 100 μM fluorescein was added to the apical compartment, and samples were collected from the basolateral compartment at defined time intervals for fluorescence quantification.

### Microscopy and Image Analysis.

COS-7 cells and mouse kidney sections were imaged using a Leica SP8 TCS microscope and a HC PL APO CS2 100×/1.4 NA oil objective. MDCKII-derived cells for transferrin uptake, endocytosis inhibition, and time-course experiments were imaged on a Nikon TiE2 microscope with a CSU-W1 spinning disk unit and a PL APO λD 60×/1.42 NA oil objective. Additional imaging of MDCKII-derived cells was performed on Zeiss LSM710 and LSM780 confocal microscopes equipped with a PL APO DIC M27 63×/1.40 NA oil objective or PL APO DIC M27 40×/1.3 NA oil objective. Image handling and analysis were done in Fiji (version 1.54p) (60).

### Statistics.

Statistics were done in GraphPad Prism (version 10.3.1). When data are presented as SuperPlots, statistics were performed on the replicate means, shown as large symbols. The small symbols represent individual datapoints (images/meshworks/TALs) within these replicates.

## Supplementary Material

Appendix 01 (PDF)

## Data Availability

All study data are included in the article and/or *SI Appendix*.
