# Supplementary material for: Control of renal calcium permeability via a tight junctional claudin switch
Source: Proc Natl Acad Sci U S A. 2025 Dec 1;122(49):e2512046122. doi: 10.1073/pnas.2512046122 (PMC12704743; doi:10.1073/pnas.2512046122)
Supplement: Supplementary file 1 — Appendix 01 (PDF) [file pnas.2512046122.sapp.pdf]

Supporting Information for

Control of renal calcium permeability via a tight junctional claudin switch

Rozemarijn E van der Veen[a], Marie Bieck[a], Nacéra Mezouar[a], Volker Haucke[a,b,c], Henrik Dimke[d,e,1], Martin Lehmann[a,1]

[a] Department of Molecular Physiology & Cell Biology, Leibniz Forschungsinstitut für Molekulare Pharmakologie (FMP), 13125 Berlin, Germany

[b] NeuroCure Cluster of Excellence, Charité Universitätsmedizin Berlin, Corporate Member of Freie Universität Berlin, Humboldt-Universität zu Berlin, and Berlin Institute of Health, Berlin 10117, Germany

[c] Faculty of Biology, Chemistry, Pharmacy, Freie Universität Berlin, 14195 Berlin, Germany

[d] Department of Cardiovascular and Renal Research, Institute of Molecular Medicine, University of Southern Denmark, 5000 Odense C, Denmark

[e] Department of Nephrology, Odense University Hospital, 5000 Odense C, Denmark

[1] Corresponding authors: MLehmann@fmp-berlin.de (ML), hdimke@health.sdu.dk (HD)

This file includes:

Figures S1 to S8
Table S1
Detailed Materials and Methods
Supplementary References

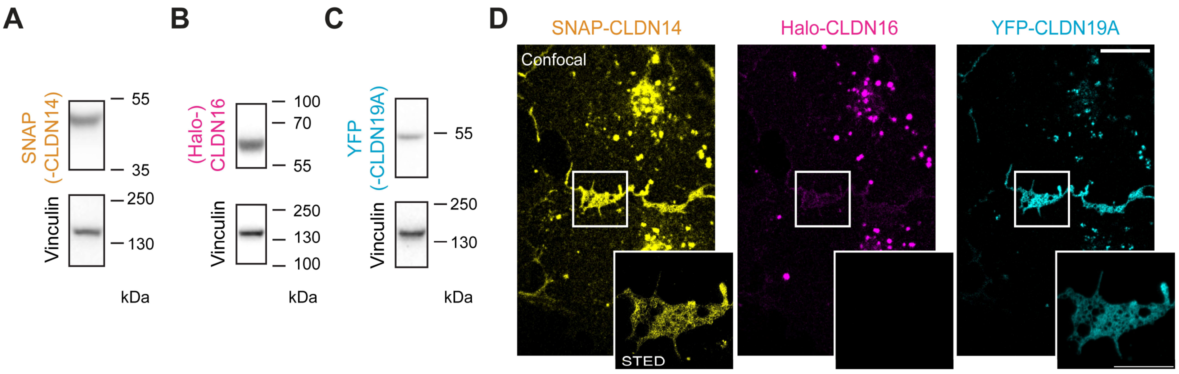

Fig. S1: Expression of CLDN14, CLDN16 and CLDN19A upon coexpression in COS-7 cells

(**A**) Immunoblot demonstrating SNAP-CLDN14 expression upon triple transfection of SNAP-CLDN14, Halo-CLDN16, and YFP-CLND19A in COS-7 cells, with vinculin as a loading control. (**B**) Immunoblot demonstrating Halo-CLDN16 expression upon triple transfection of SNAP-CLDN14, Halo-CLDN16 and YFP-CLDN19A in COS-7 cells, with vinculin as a loading control. (**C**) Immunoblot demonstrating YFP-CLDN19A expression upon triple transfection of SNAP-CLDN14, Halo-CLDN16 and YFP-CLND19A in COS-7 cells, with vinculin as a loading control. (**D**) Confocal imaging of two COS-7 cells demonstrating expression of SNAP-CLDN14 (yellow; Atto590), Halo-CLDN16 (magenta; JF646) and YFP-CLDN19A (cyan; Atto542), as well as STED imaging of the TJ-like meshwork between the cells, in which CLDN14 and CLDN19A are clearly present, but from which CLDN16 is mostly excluded. Scale bars: 10 µm (confocal) and 4 µm (STED).

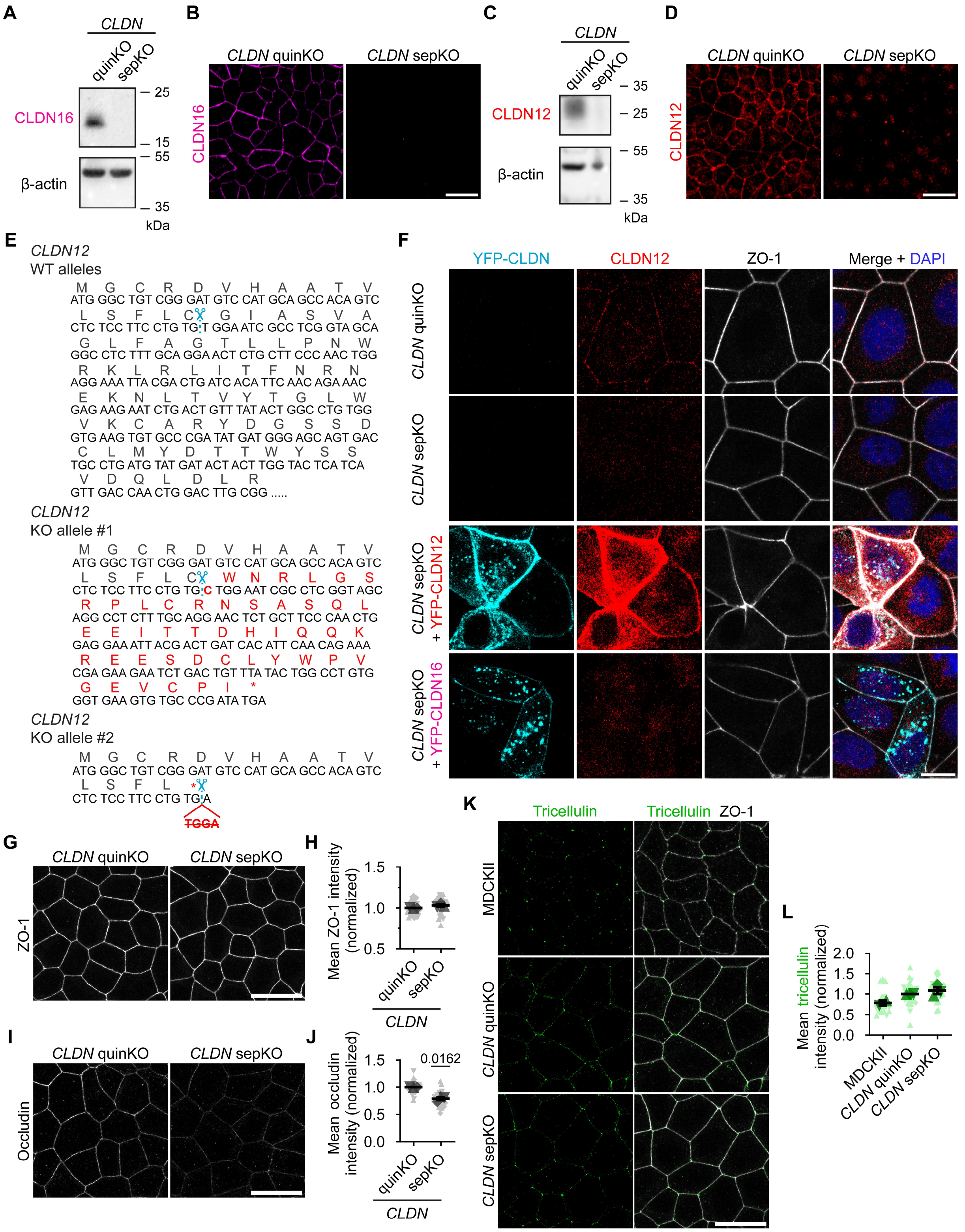

Fig. S2: Validation of CLDN16 and CLDN12 KO and subsequent characterization of *CLDN* sepKO cells

(**A**) Immunoblot of *CLDN* quinKO and sepKO cells, showing successful CLDN16 KO in the latter, with β-actin as a loading control. (**B**) Maximum intensity projections of *CLDN* quinKO and sepKO cells, stained for CLDN16 (magenta; CF488A), demonstrating its KO in *CLDN* sepKO cells. Original z-stacks: 6 to 8 images, 1 µm spacing. Scale bar: 20 µm. (**C**) Immunoblot demonstrating successful KO of CLDN12 from *CLDN* quinKO cells, resulting in *CLDN* sepKO cells, with β-actin as a loading control.

(**D**) Maximum intensity projections of *CLDN* quinKO and sepKO cells, stained for CLDN12 (red; CF640R) demonstrating its KO in *CLDN* sepKO cells. Original z-stacks: 6 to 8 images, 1 µm spacing. Scale bar: 20 µm. The cells shown are the same as in *A*. (**E**) The genomic sequences of the *CLDN12* WT alleles in *CLDN* quinKO cells and the two different KO alleles in *CLDN* sepKO cells as determined by sequencing. The Cas9 cutting site is indicated with scissors and a dotted line, and inserted/deleted bases and altered amino acids are indicated in red. KO allele #1 has a single base insertion, leading to a frameshift and a stop codon at position 62. KO allele # 2 has a 4-base deletion, which introduces a stop codon at position 16. (**F**) Validation of the CLDN12 antibody (red; CF568), which detects endogenous CLDN12 in *CLDN* quinKO cells and transfected YFP-CLDN12 (cyan) in *CLDN* sepKO cells, but does not detect anything in *CLDN* sepKO cells that are untransfected or transfected with YFP-CLDN16 (cyan). Cells were also stained for ZO-1 (gray; AF647) and DAPI (blue). Maximum intensity projection of 4 images with 1 µm spacing. Scale bar: 10 µm. (**G**) Representative maximum intensity projection images of ZO-1 (gray; AF488) levels in *CLDN* quinKO and sepKO cells. Original z-stacks: 10 images, 1 µm spacing. Scale bar: 20 µm. (**H**) Mean intensity of endogenous ZO-1 in *CLDN* quinKO and sepKO cells. Data were measured in maximum intensity projections. Replicates (n = 3) are represented by different symbol shapes. Per replicate, 10 images (small symbols) were analyzed, and the data were normalized to the average signal in *CLDN* quinKO cells. Large symbols are replicate averages, of which the mean ± SEM is shown. A one-sample *t* test was performed for the *CLDN* sepKO cells (P = 0.1249). (**I**) Representative maximum intensity projection images of occludin (gray; AF488) levels in *CLDN* quinKO and sepKO cells. Original z-stacks: 10 images, 1 µm spacing. Scale bar: 20 µm. (**J**) Mean intensity of endogenous occludin in *CLDN* quinKO and sepKO cells. Data were measured in maximum intensity projections. Replicates (n = 3) are represented by different symbol shapes. Per replicate, 10 images (small symbols) were analyzed and the data were normalized to the average signal in *CLDN* quinKO cells. Large symbols are replicate averages, of which the mean ± SEM is shown. A one-sample *t* test was performed for the *CLDN* sepKO cells. (**K**) Representative maximum intensity projection images of tricellulin (green; AF488) levels in MDCKII, *CLDN* quinKO and *CLDN* sepKO cells, costained for ZO-1 (gray; AF647). Nonspecific signal was subtracted before performing maximum intensity projections, using MDCKII cells as a reference. Original z-stacks: 10 images, 1 µm spacing. Scale bar: 20 µm. (**L**) Mean intensity of endogenous tricellulin in MDCKII, *CLDN* quinKO and *CLDN* sepKO cells. Data were measured in maximum intensity projections. Replicates (n = 2 to 3) are represented by different symbol shapes. Per replicate, 10 images (small symbols) were analyzed and the data were normalized to the average signal in *CLDN* quinKO cells. Large symbols are replicate averages, of which the mean ± SEM is shown. A one-sample *t* test was performed for the *CLDN* sepKO cells (P = 0.4).

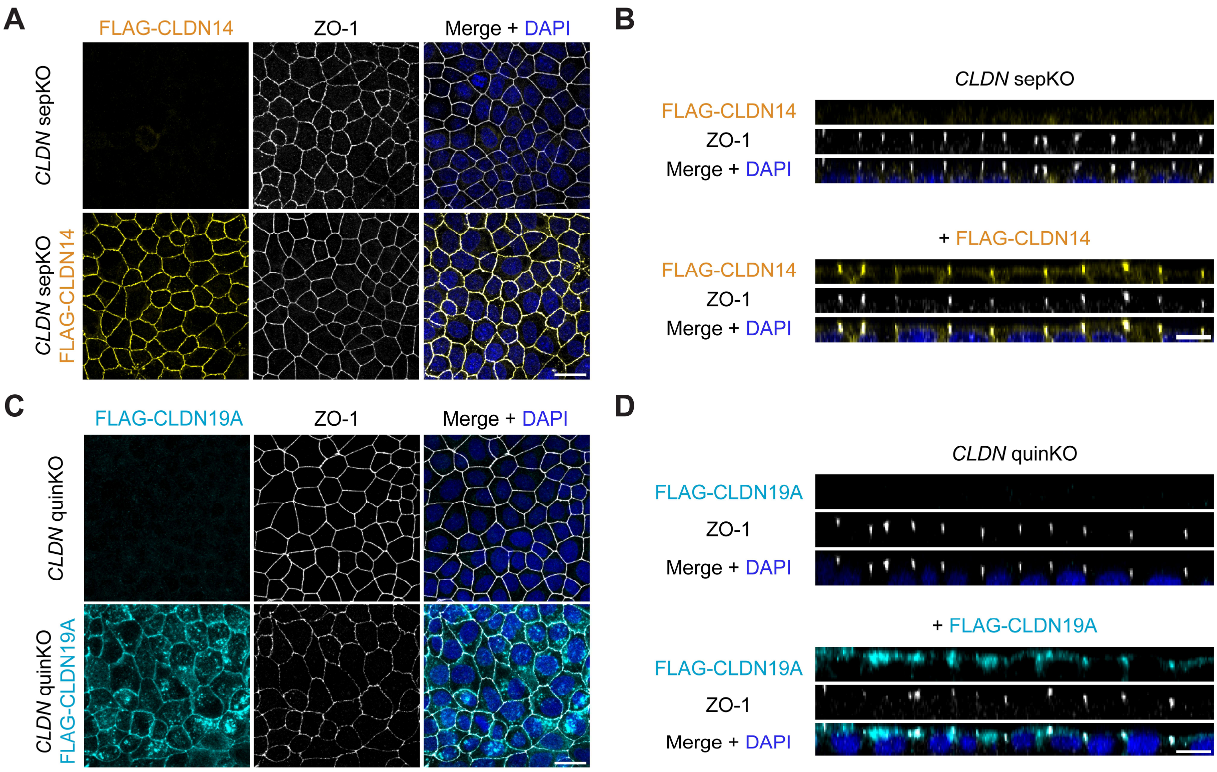

Fig. S3: Confirmation of stable FLAG-CLDN14 expression in *CLDN* sepKO cells and stable FLAG-CLDN19A expression in *CLDN* quinKO cells

(**A**) Maximum intensity projections demonstrating stable FLAG-CLDN14 expression (yellow; AF488) in *CLDN* sepKO cells, costained for ZO-1 (gray; AF647) and DAPI (blue). Original z-stacks: 7 images, 1 µm spacing. Scale bar: 20 µm. (**B**) Side view of (FLAG-CLDN14-expressing) *CLDN* sepKO cells, stained for FLAG-CLDN14 (yellow; AF488), ZO-1 (gray; AF647), and DAPI (blue). Original z-stacks: 7 images, 1 µm spacing. Scale bar: 10 µm. (**C**) Maximum intensity projections of (stable FLAG-CLDN19A-expressing) *CLDN* quinKO cells, stained for FLAG-CLDN19A (cyan; AF488), ZO-1 (gray; AF647) and DAPI (blue). Original z-stacks: 10 images, 1 µm spacing. Scale bar: 20 µm. (**D**) Side view of *CLDN* quinKO cells demonstrating stable FLAG-CLDN19A expression (cyan; AF488), costained for ZO-1 (gray; AF647) and DAPI (blue). Original z-stacks: 10 images, 1 µm spacing. Scale bar: 10 µm.

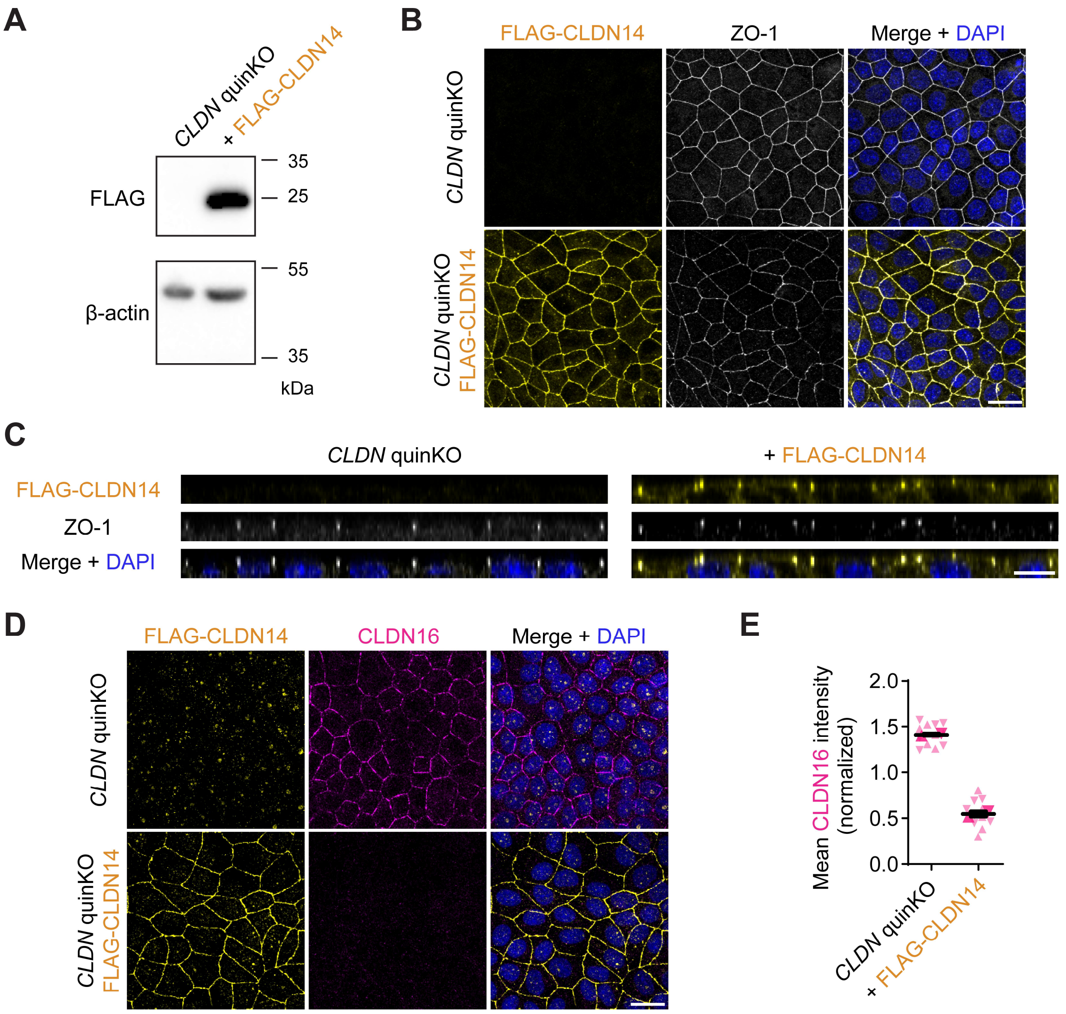

Fig. S4: Stable FLAG-CLDN14 expression in epithelial cells also leads to the displacement of CLDN16

(**A**) Immunoblot of *CLDN* quinKO cells, demonstrating stable FLAG-CLDN14 expression, with β-actin as a loading control. (**B**) Maximum intensity projections of *CLDN* quinKO cells with and without stable FLAG-CLDN14 expression (yellow; AF488). Cells were costained for ZO-1 (gray; AF647) and DAPI (blue). Original z-stacks: 7 images, 1 µm spacing. Scale bar: 20 µm. (**C**) Side views of *CLDN* quinKO cells (stably expressing FLAG-CLDN14), stained for FLAG-CLDN14 (yellow; AF488), ZO-1 (gray; AF647) and DAPI (blue). Original z-stacks: 7 images, 1 µm spacing. Scale bar: 10 µm. (**D**) Maximum intensity projections of *CLDN* quinKO cells, demonstrating that endogenous CLDN16 (magenta; AF488) disappears from their membrane upon stable FLAG-CLDN14 expression (yellow; AF647). DAPI (blue) was used to stain the nuclei. Original z-stacks: 7 images, 1 µm spacing. Scale bar: 20 µm. (**E**) Mean intensity of endogenous CLDN16 in *CLDN* quinKO cells with/without stable FLAG-CLDN14 expression. 5 to 6 images (small symbols) were analyzed per replicate (each with a different symbol shape). Intensities were measured in maximum intensity projections and were normalized to the average CLDN16 level per replicate. Replicate (n = 2) averages are shown with large symbols and their mean ± SEM is plotted.

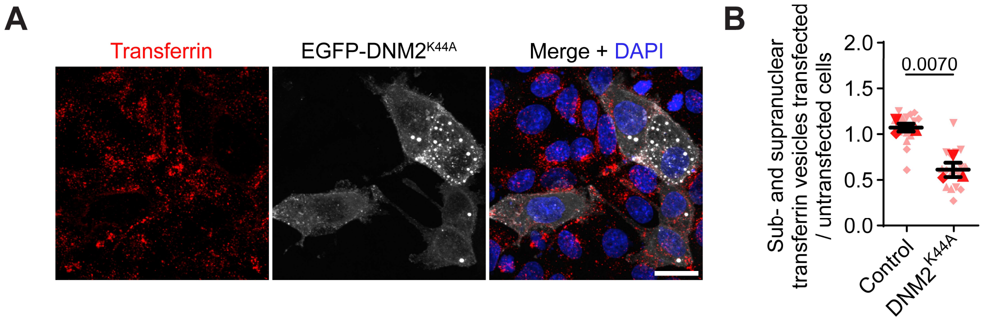

Fig. S5: DNM2^{K44A} blocks endocytosis in *CLDN* quinKO cells

(**A**) Maximum intensity projection showing a successful block of transferrin uptake (red; AF647) upon EGFP-DNM2^{K44A} transfection (gray) in *CLDN* quinKO cells. Nuclei were stained with DAPI (blue). Original z-stack: 9 images, 1 µm spacing. Scale bar: 20 µm. (**B**) The ratio of transferrin vesicles found above and below the nucleus (sub- and supranuclear) in EGFP-DNM2^{K44A} or EGFP (control) transfected *CLDN* quinKO cells versus non-transfected neighboring cells. 5 to 6 images (small symbols) were analyzed per replicate (represented by different symbol shapes). n = 3 (large symbols are replicate averages), mean ± SEM are shown, and a Student's *t* test was performed.

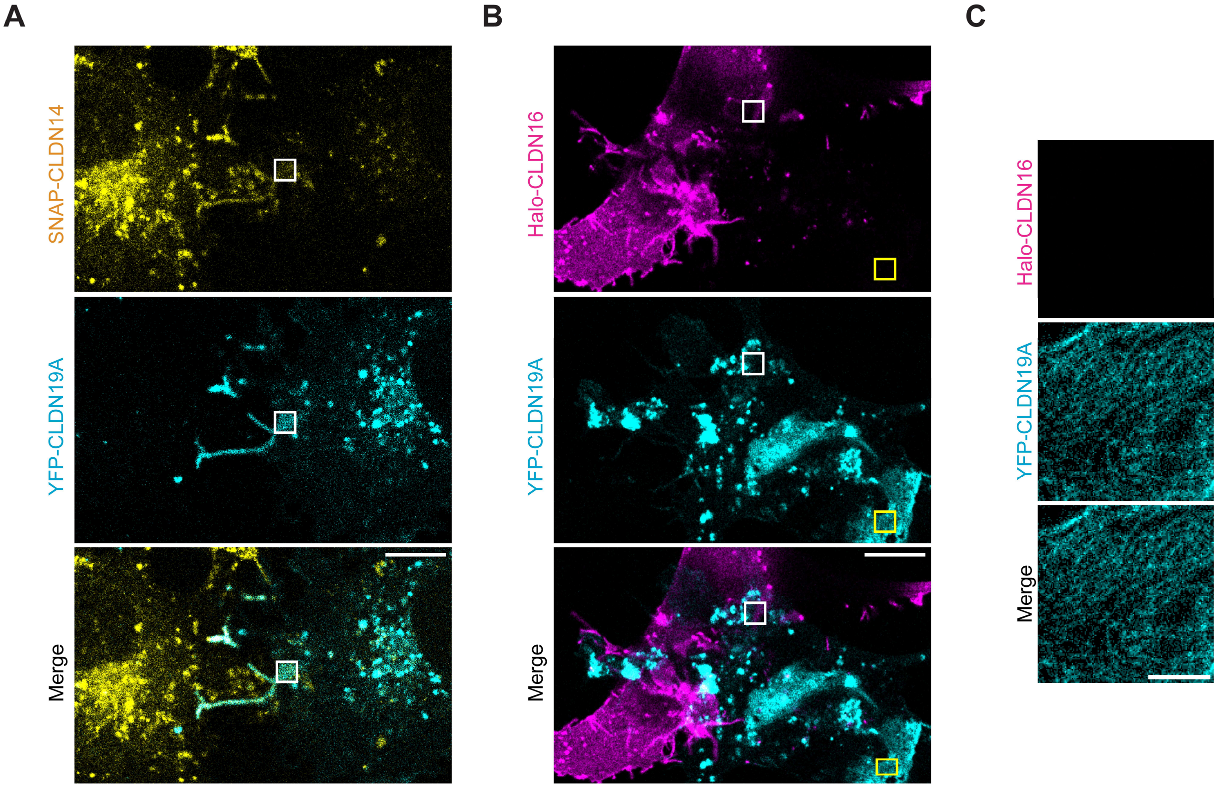

Fig. S6: Claudin expression in COS-7 cells cocultured to test claudin *trans*-interactions

(**A**) Confocal overview image of cocultured COS-7 cells expressing SNAP-CLDN14 (yellow; Atto590) or YFP-CLDN19A (cyan; Atto647N). The white box outlines the area imaged with STED as shown in Fig. 4*B*. Scale bar: 10 µm. (**B**) Confocal overview image of cocultured COS-7 cells expressing Halo-CLDN16 (magenta; Atto590) or YFP-CLDN19A (cyan; Atto647N), showing that meshworks exclusively form between CLDN19-expressing cells (e.g. yellow box; STED image of meshwork shown in *C*), with no such structures evident between cells expressing CLDN16 and CLDN19 (white box; area imaged with STED as shown in Fig. 4*C*). Scale bar: 10 µm. (**C**) STED image of a meshwork formed between COS-7 cells expressing YFP-CLDN19A (cyan; Atto647N), taken from the coculture with COS-7 cells expressing Halo-CLDN16 (magenta; Atto590) shown in *B*. Scale bar: 1 µm.

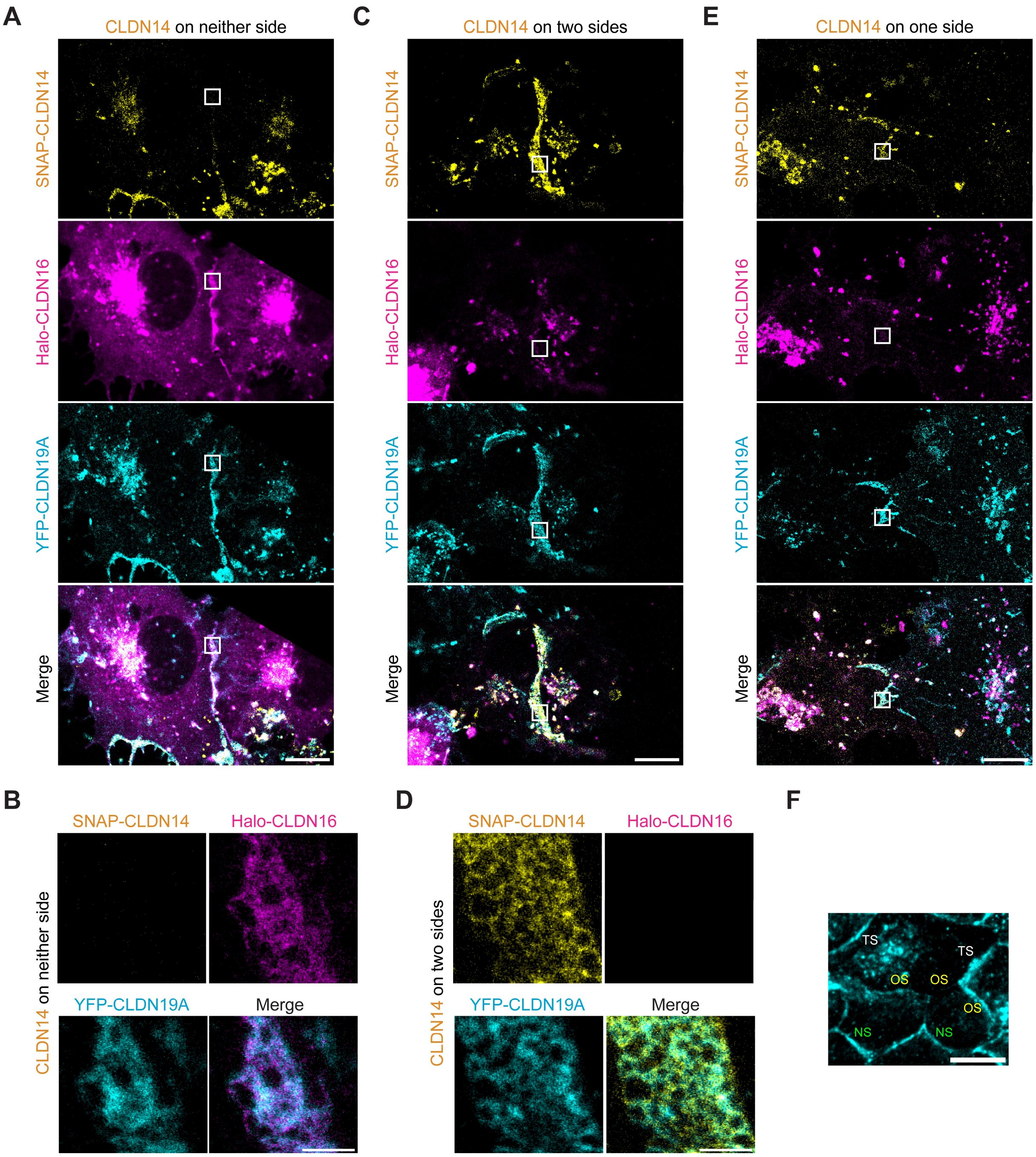

Fig. S7: CLDN16-CLDN19A meshworks in COS-7 cocultures with CLDN14 expressed on one side, two sides or neither side

(**A**) Confocal overview image of cocultured COS-7 cells each expressing Halo-CLDN16 (magenta; JF646) and YFP-CLDN19A (cyan; Atto542), but lacking SNAP-CLDN14 (yellow; Atto590). The white box outlines the area imaged with STED, as shown in Fig. *B*. Scale bar: 10 µm. (**B**) STED image of a cell-cell overlap between two COS-7 cells, which are both transfected with Halo-CLDN16 (magenta; JF646) and YFP-CLDN19A (cyan; Atto542), but not with SNAP-CLDN14 (yellow; Atto590). Scale bar: 1 µm. (**C**) Confocal overview image of cocultured COS-7 cells each expressing SNAP-CLDN14 (yellow; Atto590), Halo-CLDN16 (magenta; JF646) and YFP-CLDN19A (cyan; Atto542). The white box outlines the area imaged with STED, as shown in Fig. *D*. Scale bar: 10 µm. (**D**) STED image of a cell-cell overlap between two COS-7 cells transfected with SNAP-CLDN14 (yellow; Atto590), Halo-CLDN16 (magenta; JF646) and YFP-CLDN19A (cyan; Atto542). Scale bar: 1 µm. (**E**) Confocal

overview image of cocultured COS-7 cells each expressing Halo-CLDN16 (magenta; JF646) and YFP-CLDN19A (cyan; Atto542), but with SNAP-CLDN14 (yellow; Atto590) present in only one cell. The white box outlines the area imaged with STED, as shown in Fig. 4*F*. Scale bar: 10 µm. (**F**) Maximum intensity projection demonstrating FLAG-CLDN19A (cyan; AF647) expression in *CLDN* quinKO cells shown in Fig. 4*G*. Junctions are labelled according to expression of CLDN14 from two sides (TS), one side (OS) or neither side (NS). Original z-stacks: 10 images, 1 µm spacing. Scale bar: 10 µm.

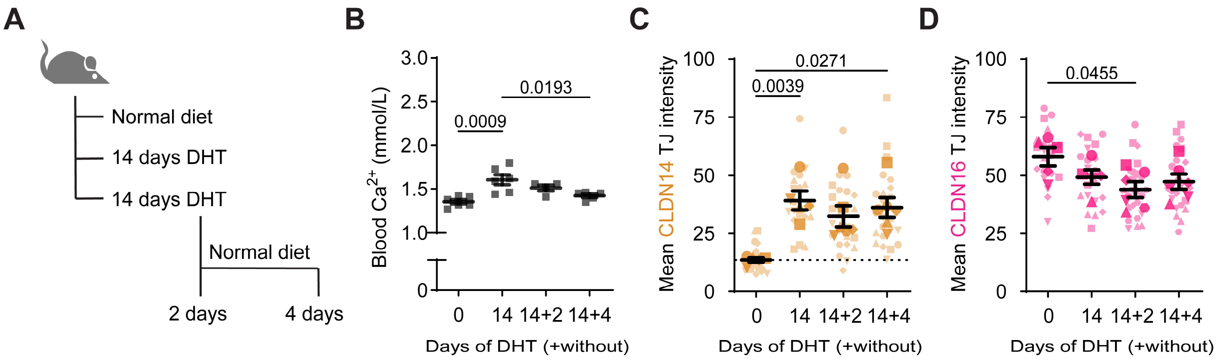

Fig. S8: Although 4 days of DHT removal reduces blood Ca^{2+} levels, this period is insufficient to reverse the claudin switch

(**A**) Experimental setup with 4 groups of mice: (1) fed with a normal diet, (2) fed with a DHT diet for 14 days, (3) fed with a DHT diet for 14 days followed by a 2-day normal diet, and (4) fed with a DHT diet for 14 days followed by a 4-day normal diet. (**B**) The effect of a DHT diet and a subsequent normal diet on blood Ca^{2+} levels in mice. n = 4 to 6 and means ± SEM are shown. A one-way ANOVA test (P = 0.0012) followed by a Tukey's multiple comparisons test was performed. P-values ≤ 0.05 are shown. (**C**) Mean CLDN14 intensity in mouse TAL TJs, as a result of a 14-day DHT diet, followed by different durations of a normal diet. 5 to 6 mice were analyzed per condition (each shown by a different symbol), 4 TALs were imaged for each (small symbols). Mouse averages are shown with large symbols, their mean ± SEM is shown, and a Kruskal-Wallis test (P ≈ 0.0043) followed by a Dunn's multiple comparisons test was performed. P-values ≤ 0.05 are shown. Absence of CLDN14 is depicted with a dotted line. (**D**) Mean CLDN16 intensity in mouse TAL TJs, as a result of a 14-day DHT diet, followed by different durations of a normal diet. 5 to 6 mice were analyzed per condition (each shown by a different symbol), 4 TALs were imaged for each (small symbols). Mouse averages are shown with large symbols, their mean ± SEM is shown, and a one-way ANOVA test (P = 0.0619) followed by a Tukey's multiple comparisons test was performed. P-values ≤ 0.05 are shown.

Table S1: Metabolic and urinary data of mice fed with a DHT diet. ns = not significant

Days DHT	0	3	7	14
**Weight (g)**				
Individual values	20.93	17.44	20.85	16.99
	21.53	19.54	18.94	17.53
	21.02	20.65	19.54	15.99
	21.80	20.85	21.70	16.84
	20.04	19.12	17.55	15.70
Mean	***21.06***	***19.52***	***19.71***	***16.61***
SEM	0.30	0.61	0.73	0.34
Holm-Šídák's multiple comparisons test (One-way ANOVA: p=0.0002)				
p vs. 0 days	-	*ns*	*ns*	*0.0001*
p vs. 3 days	-	-	*ns*	*0.005*
p vs. 7 days	-	-	-	*0.0036*
**Water intake (mL/day)**				
Individual values	6.37	6.01	6.10	5.90
	0.94	3.45	4.66	6.33
	0.87	3.46	4.92	3.58
	5.94	4.45	4.83	3.73
	3.66	2.85	5.58	2.70
Mean	***3.56***	***4.04***	***5.22***	***4.45***
SEM	1.18	0.55	0.27	0.71
Tukey's multiple comparisons test (One-way ANOVA: p=ns)				
p vs. 0 days	-	*ns*	*ns*	*ns*
p vs. 3 days	-	-	*ns*	*ns*
p vs. 7 days	-	-	-	*ns*
**Food intake (g/day)**				
Individual values	7.69	5.87	5.92	7.03
	8.75	5.31	6.64	5.13
	9.81	8.30	5.67	5.33
	9.60	7.68	6.62	4.90
	7.45	6.79	7.54	5.38
Mean	***8.66***	***6.79***	***6.48***	***5.55***
SEM	0.48	0.55	0.33	0.38
Dunn's multiple comparisons test (Kruskal-Wallis test: p~0.0106)				
p vs. 0 days	-	*ns*	*ns*	*0.0055*
p vs. 3 days	-	-	*ns*	*ns*
p vs. 7 days	-	-	-	*ns*

**Urine volume (mL/day)**				
Individual values	0.96	1.01	1.97	1.11
	0.52	0.47	0.82	1.85
	0.50	0.75	1.68	0.70
	1.54	1.12	2.09	0.44
	0.56	0.81	2.51	0.98
Mean	***0.82***	***0.83***	***1.81***	***1.02***
SEM	0.20	0.11	0.28	0.24
Holm-Šídák's multiple comparisons test (One-way ANOVA: p=0.0153)				
p vs. 0 days	-	*ns*	*0.0299*	*ns*
p vs. 3 days	-	-	*0.0299*	*ns*
p vs. 7 days	-	-	-	*ns*
**Fecal excretion (g/day)**				
Individual values	1.53	1.22	0.98	1.85
	2.25	1.25	1.44	0.67
	2.43	1.99	1.10	0.84
	2.44	1.74	1.25	0.70
	1.68	1.44	1.83	0.79
Mean	***2.07***	***1.53***	***1.32***	***0.97***
SEM	0.19	0.15	0.15	0.22
Dunn’s multiple comparisons test (Kruskal-Wallis: p~0.0240)				
p vs. 0 days	-	*ns*	*ns*	*0.0164*
p vs. 3 days	-	-	*ns*	*ns*
p vs. 7 days	-	-	-	*ns*
**Urinary Ca^{2+}/creatinine**				
Individual values	0.572	1.179	4.258	4.258
	0.684	1.037	4.774	0.339
	0.629	1.064	4.748	4.381
	0.476	1.316	1.456	1.338
	0.906	0.356	1.893	1.509
Mean	***0.653***	***0.990***	***3.426***	***2.365***
SEM	0.072	0.166	0.724	0.823
Holm-Šídák's multiple comparisons test (One-way ANOVA: p=0.0098)				
p vs. 0 days	-	*ns*	*0.0166*	*ns*
p vs. 3 days	-	-	*0.0339*	*ns*
p vs. 7 days	-	-	-	*ns*

Detailed Materials and Methods

Constructs

The Halo-CLDN16, YFP-CLDN19A, YFP-CLDN12, YFP-CLDN16, pCIG3.NB and pMD2.G plasmids used in this study were previously described (1). EGFP-Dynamin 2 K44A was a gift from Pietro De Camilli (Addgene plasmid #22301; (2)). The pEGFP-N1 construct came from Clontech (6085-1), and a FLAG-CLDN14 construct was synthesized by Absea Biotechnology Ltd. (Beijing, China), using a pEGFP-C1 plasmid as a basis, in which the EGFP was replaced by FLAG-CLDN14 through AgeI and ApaI restriction.

The SNAP-CLDN14 construct was obtained by replacing CLDN2 in a previously described SNAP-CLDN2 construct (1) with CLDN14 from the FLAG-CLDN14 construct, through restriction with FastDigest HindIII and ApaI (Thermo Fisher Scientific, FD0505 and FD1414). pLIB-CMV-FLAG-CLDN14-Puro and pLIB-CMV-FLAG-CLDN19A-Puro plasmids were generated by PCR amplification of CLDN14 and CLDN19A from the SNAP-CLDN14 and YFP-CLDN19A plasmids using the following primers: general FW 5'-TATAACCGGTATGGATTACAAGGATGACGACGATA AGCTGTACAAAAGCTTGGTACCGAGCTCGGATCCAATGG-3', *CLDN14* RV 5'-TATAGCG GCCGCCGCTCACACGTAGTCGTTCAG-3', *CLDN19A* RV 5'-TATAGCGGCCGCTCGACCGGC CGCCAGTGTGATGGATATCTTAC-3' and insertion by BshTI and NotI restriction (Thermo Fisher Scientific, FD1464 and FD0593) into a pLIB-CMV-EGFP construct also containing a puromycin selection cassette (1).

For all cloning, desired PCR and restriction products were isolated with gel electrophoresis and gel extraction (Macherey-Nagel, F40609.250) and ligated with T4 DNA ligase (Thermo Fisher Scientific, EL0016). Two different strains of *E. coli* were used for the large-scale production of DNA plasmids: HB101 cells (Promega, L2011) for pLIB constructs and self-made $CaCl_2$-competent TOP10 cells for all other constructs. Finally, DNA was isolated from selected colonies (Macherey-Nagel, 740420.50) and sequencing was used to validate the constructs (LGC Genomics GmbH, Berlin, Germany; Eurofins Genomics Germany GmbH, Ebersberg, Germany). All CLDN constructs contain the following linker sequence between their N-terminal tag and the *CLDN* sequence: base pairs – CTGTACAAAAGCT TGGTACCGAGCTCGGATCCA; amino acids - LYKSLVPSSDP. As predicted by NetPhos – 3.1 (3), the addition of tag and linker could introduce up to three phosphorylation sites in our proteins. The majority of these sites are located within the linker, with the exception of a threonine at position 2 of CLDN16. Given that the N-terminus of claudins is short and not linked to regulatory functions (4), we presume that these proposed phosphorylation sites are unlikely to alter protein function.

Cell culture and transfection

In this study, the following cell lines were used: COS-7 (ATCC CRL 1651), HEK293T (ATCC CRL 11268), MDCKII (ECACC 00062107), and MDCKII claudin quintuple knockout (*CLDN* quinKO, courtesy of Prof. Mikio Furuse, National Institute for Physiological Sciences, Japan). All cells were cultured in high glucose Dulbecco's modified Eagle's medium (DMEM) (Thermo Fisher Scientific, 11965084), supplemented with 10% (v/v) fetal bovine serum (FBS) (Gibco, Thermo Fisher Scientific, 10082147) and 100 µg/ml penicillin-streptomycin (Gibco, Thermo Fisher Scientific, 15140122) at 37 °C and in a 5% CO_2 atmosphere. Cells were regularly checked to be negative for *mycoplasma.*

For imaging experiments, cells were seeded in µ-Slide 8-well glass bottom dishes (Ibidi, 80827) or on 25 mm, #1.5H precision glass coverslips (Thermo Fisher Scientific, #CB00250RAC33MNT0). For immunoblotting, the generation of stable cell lines and coculture experiments, cells were (initially) seeded in a 6-well plate. 150.000 cells were seeded on a 12 mm, polycarbonate, 0.4 µm Millicell cell

culture insert (Merck, PIHP01250) for TJ permeability measurements, and their medium was refreshed every 2 to 3 days. When necessary, cells were transfected 24 h after seeding at 70% confluency with Lipofectamine 2000 Transfection Reagent (Thermo Fisher Scientific, 11668019), according to the manufacturer's protocol. For imaging of COS-7 cells, the μ-Slide dishes or coverslips were coated before seeding with 2% (v/v) Cultrex Reduced Growth Factor Basement Membrane Extract (Bio-techne, 353600502) in medium, and transfection was done at 30% confluency. When the COS-7 cells were cocultured, the two cell populations were detached from their respective 6-well plate wells one day after transfection, mixed, and reseeded on two coated coverslips. For timeframe experiments in stable FLAG-CLDN19a-expressing *CLDN* quinKO cells, cells were transfected at 24, 48, or 72 h, at confluencies of 30%, 45%, and 70%, respectively, and one well was left untransfected

Generation of knockout cell lines

MDCKII *CLDN* septuple knockout (sepKO) cells were generated by disrupting the *CLDN12* and *CLDN16* genes from MDCKII *CLDN* quinKO cells using the CRISPR-Cas9 system from the Zhang Lab (5). *CLDN* quinKO cells were originally created from MDCKII cells through disruption of the genes for *CLDN1*, *CLDN2*, *CLDN3*, *CLDN4* and *CLDN7* (6). gRNAs were used that target the following sequences (PAM sequences underlined): *CLDN12* 5'-<u>CCT</u>GTGTGGAATCGCCTCGGTAG-3' and *CLDN16* 5'-CACCTGGACAGACTGTTGGA<u>TGG</u>-3'. gRNAs were cloned into a pSpCas9(BB)-2A-Puro (PX459; Addgene plasmid #48139) plasmid as described in Ran *et al*. (5). In short, a FW and a RV primer coding for the gRNA were phosphorylated and annealed to each other in the presence of T4 polynucleotide kinase (Thermo Fisher Scientific, EK0032). pSpCas9(BB)-2A-Puro was digested with FastDigest BpiI (Thermo Fisher Scientific, FD1014), dephosphorylated with alkaline phosphatase (Thermo Fisher Scientific FastAP thermosensitive alkaline phosphatase, EF0651) and isolated with gel electrophoresis and gel extraction. Finally, gRNAs and backbone were ligated in a 4:1 ratio with T4 DNA ligase, DNA was amplified in self-made $CaCl_2$-competent TOP10 cells, isolated from selected colonies and validated using sequencing.

CLDN quinKO cells were seeded and transfected with the pSpCas9(BB)-2A-Puro plasmid containing the gRNA for *CLDN12*. One day later, transfected cells were selected for 24 h with 5 μg/mL puromycin (Thermo Fisher Scientific, A1113803) and cultured until 80% confluency. Cells were then seeded and transfected with the pSpCas9(BB)-2A-Puro plasmid containing the gRNA for *CLDN16*, again after one day selected for 24 h with 5 μg/mL puromycin, and then cultured up for one day until 40 % confluency. To obtain *CLDN* sepKO clones, cells were reseeded in conditioned medium in a 96-well plate at a concentration of 0.5 cell/well. After two weeks, single colonies were picked by eye, and KO of *CLDN12* and *CLDN16* was validated with immunocytochemistry and immunoblot analysis.

CLDN12 KO was further validated through isolation of genomic DNA (Macherey-Nagel, NucleoSpin Tissue XS, 740901.250), followed by PCR amplification of the *CLDN12* genomic region using the following primers: *CLDN12* FW 5'-TATAACCGGTTATTGTCCCCTCATGATTTGTCCTC-3', *CLDN12* RV 5'-TATAGCGGCCGCATATACAGGCCCCCAGCACTAG-3'. Agarose gel electrophoresis and sequencing of the PCR product showed the presence of two distinct, but similarly sized alleles. The PCR product and the previously described SNAP-CLDN14 plasmid were then digested with BshTI and NotI (Thermo Fisher Scientific, FD1464 and FD0593), excising SNAP-CLDN14 from the plasmid. The fragments were isolated by gel electrophoresis and gel extraction, then ligated with T4 DNA ligase. The ligation product was transformed into self-made $CaCl_2$-competent TOP10 cells, and multiple colonies were sequenced to determine the sequence of both KO alleles.

Generation of stable cell lines

HEK293T cells were seeded in a 10 cm dish for retrovirus production. 24 h later, at roughly 50% confluency, cells were transfected. 60 µL 2M $CaCl_2$-solution was mixed with 440 µL TE buffer (1 mM Tris, 0.2 mM EDTA, pH 8.0). In this, 10.5 µg pCIG3.NB packaging plasmid, 4.5 µg pMD2.G retroviral envelope plasmid, and 15 µg pLIB-CMV-FLAG-CLDN-Puro-plasmid were combined and incubated at RT for 5 min. Under slight agitation, this solution was added dropwise into 500 µL 2x HBS solution (50 mM Hepes, 280 mM NaCl, 1.5 mM Na_2HPO_4, pH 7.05), and the combination was incubated another 20 min at RT before it was used for transfection. After 24 h, the medium was refreshed, and after 3 to 4 and 5 to 6 days, the virus-containing medium was collected, spun down at 720 g for 5 min, and the supernatant was used for cell transduction. *CLDN* quinKO and sepKO cells were transduced 48 h after seeding at a confluency of 50 to 60% with a mixture of 50% virus-containing supernatant and 50% fresh medium. 2 to 3 days after transduction, cells were selected with puromycin. *CLDN* quinKO cells were selected for 2 to 3 days with 3 µg/mL puromycin; *CLDN* sepKO cells were selected for 4 to 5 days with 5 µg/mL puromycin. Cells were used as a mixed population; no clones were picked. Finally, all cells were maintained up to a maximum of 80% confluency in 2 µg/mL puromycin, which was refreshed every 2 to 3 days.

SNAP- and Halo-tag labeling

Various SNAP (BG) and Halo (CA) substrates were used to label proteins in this study. BG-Atto590, BG-JF646, CA-Atto590 and CA-JF646 were synthesized from BG-NH_2 (New England Biolabs Inc., S9148S) or CA-NH_2 (Promega, P6711), and Atto590-NHS (Sigma Aldrich, 79636) or JF646-NHS (Tocris, 6148), as described by Bottanelli *et al.* (7). BG-JFX554 was a gift from Luke Lewis (Janelia Farm). Labeling was done 24 h after (the last) transfection, before fixation or imaging. Cells were labelled with 1 µM (for CA-substrates and BG-JFX554) or 2 µM (for BG-Atto590 and BG-JF646) substrate for 30 min to 1 h at 37 °C under a 5% CO_2 atmosphere, and cells were extensively washed afterwards. STED samples were left to wash out excess substrate for another 30 min.

Fixation and immunocytochemistry (ICC)

COS-7 cells were fixed with pre-warmed 4% (w/v) paraformaldehyde and 4% (w/v) sucrose in PBS for 20 min at 37 °C and permeabilized with 0.2% (v/v) Triton X-100 in PBS for 5 min at RT. MDCKII(-derived) cells were fixed 24 h after (the last) transfection or when they reached at least 80% confluency. Fixation was done with ice-cold 100% ethanol for 15 min at -20 °C. Cells were blocked with blocking buffer (BB; 6% (v/v) normal goat serum, 1% (w/v) bovine serum albumin, and 0.05% (v/v) Tween-20 in PBS) for 30 min at RT. COS-7 cells were incubated with primary antibodies for 1 h at RT in BB, MDCKII(-derived) cells for 1 to 3 h. Excess antibodies were thoroughly washed away with PBS, after which cells were incubated with secondary antibodies in BB for 30 min at RT. In the last 10 min of the final antibody incubation (except for STED samples), 4′,6-diamidino-2-phenylindole (DAPI, 1:5000, Thermo Fisher Scientific, 62248) was added, and finally, cells were thoroughly washed with PBS. Coverslips were mounted in ProLong Gold Antifade Reagent (Thermo Fisher Scientific, P36930).

Animal experiments

The animal experiments were conducted in accordance with Danish Law under animal experimental permits #2014-15-0201-00043 and #2019-15-0201-01629. Access to drinking water and food was unlimited. Standard rodent diet contained 0.7% Ca^{2+}, 0.2% Mg^{2+}, and 600 IU Vitamin D3 (Altromin Spezialfutter GmbH & Co. KG, TD.00374).

To induce hypercalcemia, the vitamin D analog dihydrotachysterol (DHT) was used. DHT (Sigma Aldrich, D9257-50mg) was added to the diet by dissolving 1 mg in 250 µL ethanol, after which it was mixed with water and added to 100 g of rodent diet. To test the effect of different durations of the DHT

diet, 20 female FVB/N mice (Janvier Labs, 12 weeks old) were randomly assigned to 4 groups (n=5) that were maintained on a normal diet or received 3, 7, or 14 days of DHT diet. On the first day of the experiment, the 14-day diet was started, whereas the 7- and 3-day diets were started later so that all mice were of the same age at the end of the experiment. Towards the end of the study, mice were placed in metabolic cages for 3 days to acclimatize, and measurements were made on the final day thereafter for all mice.

To test the reversibility of the DHT effect, 22 female FVB/N mice (Janvier Labs, 13 weeks old) were randomly divided into 4 groups. *i)* 5 mice were kept on a normal diet, *ii)* 5 mice were kept on a DHT diet for 14 days, *iii)* 6 mice were kept on a DHT diet for 14 days and then maintained on a regular diet for 4 days, and *iv)* 6 mice were kept on a DHT diet for 14 days and then maintained on a regular diet for 2 days. Mice were all placed in metabolic cages for acclimation for 3 days towards the end of the experiment, and measurements were made on the final day thereafter for all mice.

At the end of the experiments, mice were anesthetized with 1.5% isoflurane (Nicholas Piramal Limited) and opened with a large laparotomy. Blood samples were taken from the vena cava, and cervical dislocation was used to euthanize the mice. Kidneys were isolated and cut in half. One half was fixed in 4% paraformaldehyde in PBS at 4 °C overnight for immunohistochemistry, the other half frozen in liquid nitrogen and stored at -80 °C until RNA extraction.

Biochemical measurements

Blood ionized Ca^{2+} concentrations were measured using an ABL835 analyzer (Radiometer, Copenhagen, Denmark). Ca^{2+} was measured in urine using ion chromatography (Dionex Aquion Ion Chromatography System, Thermo Fisher Scientific, Denmark) and an autosampler, as previously described (8, 9). In brief, samples were diluted in double distilled water (ddH_2O) and eluted in 20 mM Methanesulfonic acid, with Dionex cation-I standards (Dionex, Thermo Fisher Scientific) used for reference. Urine creatinine concentrations were measured using an ABX Pentra Creatinine 120 CP kit (HORIBA ABX SAS, Montpellier, France) (10).

RNA extraction and quantitative PCR

Total RNA was extracted from half a mouse kidney using TRIzol reagent (Invitrogen,15596026) following the manufacturer's instructions as described previously (9, 11). Genomic DNA was removed by DNase I treatment (Thermo Fisher Scientific, EN0521b), and cDNA synthesized using the iScript cDNA synthesis kit (BioRad, 1708891). Quantitative PCR was then performed using the iTaq Universal SYBR Green Supermix (BioRad, 1725121) and a CFX Opus 384 Real-Time PCR system (BioRad). Gene expression was analyzed using the $2^{-\Delta\Delta Ct}$ method. Expression was normalized to the housekeeping gene that codes for 60S ribosomal protein L41 (*Rpl41*). The following primers were used: *Cldn14* FW 5'-CCCAAAGGACCAATGATG-3', *Cldn14* RV 5'-TTTTTCACGCTTCCAAGAC-3', *Cldn16* FW 5'-GCCATATTCTCCACTGGGTT-3', *Cldn16* RV 5'-AGTCATCAGCGTTCACCATC-3', *Cldn19* FW 5'-CAGGTGCAATGCAAACTCTACG-3', *Cldn19* RV 5'-ACTTCATGCCCACGACACTG-3', *Rpl41* FW 5'-TCTTAGCGCCATCTTCCTTG-3', *Rpl41* RV 5'-AGCATCCCTCACTTCTGCTC-3'. One sample exhibited very low housekeeping gene expression and resulting expression gene expression ratios (*Cldn14*, *Cldn16* and *Cldn19 to Rpl41*) were flagged as outliers using the ROUT method in GraphPad Prism (version 10.3.1) and therefore excluded.

Immunohistochemistry (IHC)

Fixed kidneys were dehydrated with graded ethanol and Tissue-Tek Tissue Clear (Sakura Finetek) and were embedded in paraffin on a Tissue-Tek Vacuum Infiltration Processor 6 (Sakura Finetek). A HM

355S Automatic Microtome (Thermo Fisher Scientific) was used to section the tissue at 5 µm. Sections were rehydrated using Tissue-Tek Tissue Clear (Sakura Finetek) first and a series of graded ethanol after. Antigens were retrieved by boiling the section 10 min in Tris-EGTA buffer (10 mM Tris, 0.5 mM EGTA in ddH_2O, pH 9.0) in a microwave. 50 mM NH_4Cl in PBS was used to block free aldehyde groups, and 0.6% (v/v) H_2O_2 was added to block endogenous peroxidase enzymes. Sections were then incubated with primary antibodies overnight at 4 °C and washed with 0.05% (v/v) Tween-20 in PBS. Secondary antibody staining was done for 60 min at RT in 0.05% (v/v) Tween-20, and unbound primary and secondary antibodies were removed by boiling the sections for 10 min in the microwave in Tris-EGTA buffer. Biotinylated antibody was incubated overnight at 4 °C in 10% (v/v) fetal calf serum (FCS) in PBS. 0.05% (v/v) Tween-20 in PBS was used to wash away unbound antibodies. Endogenous biotin was not blocked before sections were incubated with biotinylated antibody. 2.5 µg/mL AF594-conjugated streptavidin (Invitrogen, S11227) was added in 10% (v/v) FCS in PBS and incubated for 2 h at RT. Sections were mounted in ProLong Gold Antifade Reagent (Thermo Fisher Scientific, P36930).

Cell lysis and immunoblotting (IB)

Cells were lysed when confluent or 24 h after transfection. After one wash with ice-cold PBS, cells were lysed on ice with 100 µL ice-cold lysis buffer (1% Triton X-100, 20 mM Hepes, pH 7.4, 130 mM NaCl, 10 mM NaF, and 0.03% protease inhibitor cocktail) and incubated under constant agitation for 30 min on ice. Samples were centrifuged for 20 min at 4 °C at 17,000 g, and the supernatant was collected. Protein concentration was determined with a Bradford assay by measuring the OD595 on a photometer (BioPhotometer plus, Eppendorf). Finally, protein lysates were denatured at 95 °C for 5 min in 6x SDS sample buffer (0.375 M Tris–HCl (pH 6.8), 10% (w/v) SDS, 60% (v/v) glycerol, 0.6 M DTT, 0.06% (w/v) bromophenol blue).

25-30 µg protein was size-separated for 2 h at 100 V on a NuPAGE 4-12% Bis-Tris gel (Invitrogen, NP0336BOX) in NuPAGE MES SDS-buffer (Invitrogen, NP0002). Proteins were transferred to a nitrocellulose membrane (Cytiva, 1060004) for 90 min at 110 V in transfer buffer (10% (v/v) methanol, 25 mM Tris–HCl (pH 7.6), 192 mM glycin) on ice. Membranes were blocked for 1 h at RT with 5% (w/v) milk in TBS-T (0.1% (v/v) Tween-20, 0.01 M Tris-Base, and 0.07 M NaCl, pH 7.6). Primary antibodies were incubated overnight at 4 °C in 3% (w/v) bovine serum albumin in TBS-T. Unbound antibodies were washed away with TBS-T, after which membranes were incubated for 1 h at RT with horseradish peroxidase (HRP)-conjugated secondary antibodies in 5% (w/v) milk in TBS-T. After washing off unbound antibodies with TBS-T, HRP substrate (SuperSignal™ West Pico PLUS Chemiluminescent Substrate, Thermo Fisher Scientific, 34580) was added. Proteins were visualized on a ChemiDoc XRS+ imaging system (BioRad) controlled by the Image Lab software (version 6.1.0) and protein levels were quantified using the Image Lab software as well.

Antibody purification and biotinylation

For antibody purification, 35 mL of mice hybridoma supernatant was mixed with 9 mL of 25x PBS (pH 8), spun down for 30 min at 4 °C at 20,000 g, and the supernatant was collected. A column containing protein A (MabSelect SuRe antibody purification resin, Cytiva) was washed with 10 mL 5x PBS (pH 8), the supernatant was loaded and the column was washed with 10 mL 5x PBS (pH 8) once more. Elution with 0.5% (w/v) citric acid was performed in two steps, using 500 µL and 2 mL solution, respectively. Only the latter was collected and neutralized with 500 µL neutralization buffer (0.6 M $NaHCO_3$, 0.25 M $NaCO_3$, 0.1 M $Na_2HPO_4 \cdot 2H_2O$). 10 mM EZ-Link Sulfo-NHS-LC-LC-Biotin (Fisher Scientific, 10740625) was dissolved in sterile H_2O and added to the purified antibodies in 20-fold molar excess. The reaction was incubated for 30 min at RT, after which 50 µL 1M lysine was added to stop the reaction.

Antibodies

The following primary antibodies were used in this study: rabbit anti-CLDN12 (ICC - 1:100; IB – 1:200, IBL-America, 18801), mouse anti-CLDN16 (ICC - 1:100; IB – 1:50, (12, 13)), mouse anti-FLAG (ICC - 1:200; IB – 1:1000, Sigma Aldrich, F3165), rabbit anti-ZO-1 (ICC - 1:500; IHC – 1:100, Thermo Fisher Scientific, 61-7300), mouse anti-ZO-1 (ICC – 1:100, Invitrogen, 33-9100), mouse anti-occludin (ICC – 1:250, Invitrogen, 33-1500), rabbit anti-tricellulin (ICC – 1:600, Invitrogen, 700191), mouse anti-GFP (ICC - 1:500, Invitrogen, A-11120), mouse anti-CLDN14 (IHC - undiluted culture supernatant, (12)), mouse anti-β-actin (IB - 1:10000, Sigma-Aldrich, A5441), rabbit anti-SNAP (IB – 1:500, New England Biolabs Inc., P9310S), rabbit anti-GFP (IB – 1:2000, Abcam, ab6556), rabbit anti-FLAG (IB – 1:500, Millipore, F7425) and mouse anti-vinculin (IB - 1:500, Sigma Aldrich, V9264).

Secondary antibodies used were: goat anti-rabbit AF488 (ICC - 1:200, Thermo Fisher Scientific, A11034), goat anti-mouse AF488 (ICC - 1:200, Invitrogen, A11029), goat anti-rabbit AF647 (ICC - 1:200, Thermo Fisher Scientific, A21244), goat anti-mouse AF647 (ICC - 1:200, Thermo Fisher Scientific, A21236), donkey anti-mouse CF488A (ICC - 1:1000, Biotium, 20014), donkey anti-mouse CF568 (ICC - 1:200, Biotium, 20105-1), donkey anti-rabbit CF568 (ICC - 1:200, Biotium, 20098-1), donkey anti-rabbit CF640R (ICC - 1:1000, Biotium, 20178-1), goat anti-mouse AF488 (IHC - 1:500, Thermo Fisher Scientific, A21121), donkey anti-mouse Atto542 (ICC - 1:200, created out of donkey anti-mouse IgG (H+L) (Jackson ImmunoResearch Ltd., 715-005-151) and Atto542-NHS (ATTO-TEC, AD542-31)), donkey anti-rabbit Atto647N (IHC - 1:500, created out of donkey Anti-Mouse IgG (H+L) (Jackson ImmunoResearch Ltd., 711-005-151) and Atto647N-NHS (ATTO-TEC, AD647N-31)), HRP-conjugated goat anti-mouse (IB - 1:5000, Jackson, 115035003) and HRP-conjugated goat anti-rabbit (IB - 1:5000, Jackson, 111035003).

Alpaca anti-GFP Atto647N (ICC - 1:200, Chromotek, gba647n) was used for YFP boosting, and included in the primary antibody incubation step. Finally, biotinylated mouse anti-CLDN16 (IHC – 1:50, (12, 13)) was used.

Transepithelial electrical resistance (TER), fluorescein and ion permeability measurements

For paracellular permeability measurements, an Ussing chamber, specifically designed to fit Millicell cell culture inserts, was used (14). To prevent precipitation of Ca^{2+} with HCO_3^- in the Ringer's solution, bicarbonate-free Ringer's solution (1.2 mM $CaCl_2$, 3 mM HEPES, 5.4 mM KCl, 1 mM $MgCl_2$, 140 mM NaCl, pH 7.4) was used. Normally, we heat the Ussing chamber to 37 °C and bubble our Ringer's solution with 5% CO_2 in O_2 and the CO_2 equilibrates with HCO_3^- to ensure a stable pH. This combination of gases is used as it is a standard in literature (15-19), as it helps ensure adequate oxygenation even if there are unstirred layers (20). As our Ringer's solution is bicarbonate-free, we switched to bubbling with 100% O_2, thus staying as close to our standard as possible to obtain a stable pH and ensure adequate oxygenation. TER values were measured with 5 mL Ringer's solution on each side of the filter. Subsequently, 5 mL bicarbonate-free mannitol solution (1.2 mM $CaCl_2$, 3 mM HEPES, 5.4 mM KCl, 1 mM $MgCl_2$, 280 mM mannitol, pH 7.4) was added on the apical side, and 5 mL Ringer's solution on the basolateral side. The resulting transepithelial voltage was recorded after 10 min, and from this, the absolute Na^+ and Cl^- permeabilities were calculated as previously described (15).

Next, 5 mL bicarbonate-free Ca^{2+} solution (94.5 mM $CaCl_2$, 3 mM HEPES, 5.4 mM KCl, 1 mM $MgCl_2$, pH 7.4) or 5 mL bicarbonate-free Mg^{2+} solution (1.2 mM $CaCl_2$, 3 mM HEPES, 5.4 mM KCl, 94.3 mM $MgCl_2$, pH 7.4) was added to the basolateral side, and 5 mL Ringer's solution to the apical side. After 10 min, the transepithelial voltage was recorded. The basolateral compartment was then thoroughly washed by removing 5 mL solution and replenishing with 5 mL Ringer's solution 5 times. Next, 5 mL of Mg^{2+} or Ca^{2+} solution (whichever was not used in the first round) was added to the basolateral side

and 5 mL Ringer's solution to the apical side, and the voltage was read out once more after 10 min. From the voltages from both rounds, the absolute Ca^{2+} and Mg^{2+} permeabilities were calculated as described previously (15).

The basolateral side was again washed 5 times through the replacement of 5 mL solution with 5 mL fresh Ringer's solution. Finally, the solutions on both sides were equilibrated by the removal of 5 mL from the basolateral side and the addition of 5 mL mannitol solution. After application of a voltage clamp, 15 µL of 100 mM fluorescein was added apically. Every 5 min for 15 min, a small sample was taken from the basolateral side and replenished with a 1:2 mixture of mannitol and Ringer's solution. Samples were collected in a 96-well plate (Corning, 3365), and concentrations were determined by measuring 525 nm emission after 490 nm excitation on a Tecan Infinite 200 plate reader (Tecan Trading AG).

Transferrin uptake

One day after transfection, cells were serum-starved for 2 h in DMEM without supplements at 37 °C and 5 % CO_2. They were then incubated with 25 µg/mL transferrin-AF647 (Thermo Fisher Scientific, T23336) in DMEM for 30 min - 1 h at 37 °C and 5 % CO_2. Unbound transferrin was thoroughly washed away with DMEM, and cells were fixed with 4% (w/v) paraformaldehyde and 4% (w/v) sucrose in PBS for 10 min at RT. Nuclei were then stained by 10 min incubation at RT with DAPI (1:5000) in PBS, and cells were thoroughly washed with PBS.

Imaging of COS-7 cells and mouse kidney sections

COS-7 cells and mouse kidney sections were imaged with a Leica SP8 TCS microscope (Leica Microsystems) equipped with a pulsed white-light excitation laser (~80 ps pulse width, 80 MHz repetition rate; NKT Photonics), STED depletion lasers at 592 and 775 nm, and hybrid detectors (HyDs). The microscope was controlled with the Leica LAS X software, and a HC PL APO CS2 100x/1.4 NA oil objective (Leica Microsystems), 518 F Zeiss oil, and a time gate of 0.3 - 6 ns were used. Images were taken with a scanning speed of 400 Hz and 1024 x 1024 pixels. During sequential imaging, confocal images were taken before STED images.

COS-7 cells were imaged at RT in PBS (µ-Slide dishes) or as a mounted sample (coverslips). The following sequential fluorophore settings were used to image TJ-like meshworks in COS-7 cells: JF646/Atto647N (Ex.: 646 nm; Em.: 656–750 nm), Atto590 (Ex.: 590 nm; Em.: 600-640 nm), Atto542 (Ex.: 540 nm; Em.: 550-590 nm). During STED, all fluorophores were depleted with the 775 nm laser; a 6x zoom was used, resulting in a pixel size of 18.94 nm, and a line average of 16 was used. Whole-cell confocal images were imaged with a line average of 1 and without zoom, resulting in a 113.64 nm pixel size.

Kidney TAL images were taken with the following sequential fluorophore settings: Atto647N (Ex.: 646 nm; Em.: 656–750 nm), AF594 (Ex.: 590 nm; Em.: 600-640 nm), AF488 (Ex.: 488 nm; Em.: 498-538 nm). Confocal images of TALs from mice that had 0, 3, 7, or 14 days of DHT diet were imaged with a 2x zoom, resulting in a pixel size of 56.82 nm and a line average of 4. Here, 6-image z-stacks with 1 µm spacing were taken. For STED imaging of TJs in the same sections, the 775 nm laser was used to deplete Atto647N and AF594, and the 595 nm laser for the depletion of AF488. A line average of 16 and a 6x zoom were used, resulting in an 18.94 nm pixel size. Corresponding confocal images were taken with a line average of 2. Finally, confocal images of TALs from mice that were part of the DHT removal experiment were taken with a zoom of 3, resulting in a pixel size of 37.88 nm. In this case, z-stacks of 5 images with 1 µm spacing were taken.

Imaging of MDCKII-derived cells

MDCKII-derived cells were imaged at RT in PBS. Images of transferrin uptake, endocytosis inhibition, and timeframe experiments in MDCKII-derived cells were acquired on a Nikon TiE2 equipped with a confocal spinning disk unit (CSU-W1, Yokogawa) with a PL APO λD 60x/1.42 NA oil objective without additional magnification and Nikon immersion Oil Type F / 30cc. The microscope is equipped with two sCMOS cameras (pco.edge, 4.2bi, 6.5 µm/pixel, 2048x2048 pixel) and controlled with NIS-Elements software (Nikon). Images were taken with the following sequential fluorophore settings: DAPI (Ex.: 405 nm; Em.: 420-460 nm), EGFP/AF488 (Ex.: 488 nm; Em.: 500-550 nm), JFX554 (Ex.: 561 nm; Em.: 574-626 nm), AF647 (Ex.: 638 nm; Em.: 670-746 nm). Images were taken full-frame 2048 x 2048 pixel or as 2x2 large images with 15% overlap over a z-range of 7 to 17 µm with 1 µm spaced z-steps and 108nm pixel size.

All other images of MDCKII-derived cells were acquired with an LSM710 (Carl Zeiss Microscopy) or LSM780 (Carl Zeiss Microscopy), which are both equipped with photomultiplier tubes and a spectral detector. The LSMs were controlled by Zeiss Zen Black software (Carl Zeiss Microscopy) and 518 F Zeiss oil was used. For CLDN12 antibody verification and characterization of *CLDN* sepKO cells a PL APO DIC M27 63 × /1.40 NA oil objective (Carl Zeiss Microscopy) was used; for other experiments a PL APO DIC M27 40x/1.3 NA oil objective was used. Images were taken with 512 x 512 or 1024 x 1024 pixels and a line average of 1 or 2. For two-color imaging, the sequentially imaged fluorophores were: DAPI (Ex.: 405 nm; Em.: 414–485 nm), AF488 (Ex.: 488 nm; Em.: 493–535 nm). For three-color imaging, the sequentially imaged fluorophores were: DAPI (Ex.: 405 nm; Em.: 415–480 nm), AF488 (Ex.: 488 nm; Em.: 492–535/578 nm), AF647 (Ex.: 633 nm; Em.: 638–752 nm). For four-color imaging, these were: DAPI (Ex.: 405 nm; Em.: 415–480 nm), YFP/AF488/CF488A (Ex.: 488 nm; Em.: 492–561 nm), CF568/AF594 (Ex.: 561 nm; Em.: 570-630 nm), AF647/JF646/CF640R (Ex.: 633 nm; Em.: 638–752 nm). Z-stacks of 7 to 10 images with a 1 µm spacing were obtained.

Image analysis

Image handling and analysis were done in Fiji (version 1.54p) (21). All z-stack images were converted to a single plane image before analysis through maximum intensity projection.

Colocalization

Pearson's correlation coefficients were determined as a measure of colocalization with the Fiji plugin Coloc 2, using 10 Costes randomizations and Costes threshold regression. In the case of colocalization in TJ-like meshworks in COS-7 cells, images were cropped to have the whole cell-cell overlap in focus and to avoid vesicles. Here, a point spread function of 2 pixels (38.8 nm) was used. To quantify colocalization within *CLDN* quinKO cell TJs, a 15-pixel (1.625 µm) wide line was drawn covering the TJ. In this region colocalization was measured with a point spread function of 2 pixels (217 nm).

Comparison of ZO-1, occludin and tricellulin in CLDN quinKO and sepKO cells

The mean intensity of endogenous ZO-1, occludin and tricellulin was measured in entire maximum intensity projection images. To obtain reliable tricellulin values, nonspecific signal was subtracted before generating the maximum intensity projections, using MDCKII cells as a reference.

CLDN TJ intensity in CLDN quinKO-derived cells

CLDN14- (and EGFP-)expressing cells were selected manually. When analyzing the effect of SNAP-CLDN14 expression on CLDN16 TJ intensity in *CLDN* quinKO cells, TJs were selected by thresholding the ZO-1 channel. The overall mean CLDN16 intensity in TJs between CLDN14-expressing cells and the overall mean CLDN16 intensity in TJs between non-expressing cells was measured. In each image, mean background intensity was measured in three 10 x 10 µm ROIs and the average background

intensity was subtracted. For timeframe experiments in FLAG-CLDN19a-expressing *CLDN* quinKO cells as well as experiments with EGFP(-DNM2^{K44A}), TJs were selected manually by drawing a 10-pixel (1.083 µm) wide line over them, and their mean CLDN16 (and CLDN14) intensity was recorded. For timeframe experiments, CLDN16 mean background intensities were measured in 6.5 x 6.5 µm ROIs in 5 images per timepoint. CLDN14 mean background intensity was measured in 5 images from day 0 (no CLDN14 expression). Average background intensity was subtracted per experiment.

CLDN16 intensity in junctions with one-sided (OS), two-sided (TS) and neither-sided (NS) CLDN14 expression

In each image, lines were drawn over all TJs between a CLDN14-expressing and non-expressing cell, *i.e.* one-sided (OS) junctions. An equivalent number of lines was drawn across TJs between two non-expressing cells, *i.e.* neither-sided (NS) junctions. Finally, lines were drawn across all TJs between two CLDN14-expressing cells, *i.e.* two-sided (TS) junctions. 5-pixel (1.038 µm) lines were used in images acquired with the LSM710. 10-pixel (1.083 µm) lines were used for Nikon-CSU-W1 images. Within each line, the mean CLDN16 intensity was measured. In the case of normal *CLDN* quinKO cells, mean background intensity was measured in three 10 x 10 µm ROIs per image and the overall average background intensity was subtracted per experiment. In the case of *CLDN* quinKO cells expressing FLAG-CLDN19A, CLDN16 mean background intensities were measured in 6.5 x 6.5 µm ROIs in 5 images per timepoint and the average background intensity was subtracted per experiment.

CLDN intensity in the TAL TJ

To quantify confocal kidney TAL images, the tubule of interest was manually selected (based on the ZO-1 channel), and the signal of neighboring tubules was cleared. An outline of the TJ was created by thresholding the ZO-1 channel, and its area was recorded. The threshold was optimized per experiment based on the ZO-1 staining in multiple images from different conditions. Sections with poor staining were filtered out by eliminating sections with an average TJ outline below 12.5 µm^2. In the other sections, the mean intensity within the TJ outline in the CLDN14 and CLDN16 channels was measured.

Transferrin uptake

EGFP-expressing cells were selected manually. The DAPI channel was subjected to a 10-pixel (1.083 µm) median filter, a "Triangle white" auto threshold, and another 5-pixel (0.542 µm) median filter. Nuclei were separated with the "Watershed" function and detected using the "Analyze particles" function (Size: 50 µm^2-Infinity; Circularity: 0.50-1.00). The transferrin channel was subjected to a Gaussian Blur (Sigma: 1 pixel/108 nm). After this, transferrin vesicles within the determined nuclear outlines (*i.e.* located above or below the nucleus) were counted using the "Find Maxima" function (Prominence > 20). We focused on vesicles in this region to prevent overlap with neighboring non-transfected cells. Nuclei as well as sub- and supranuclear transferrin vesicles were counted in the EGFP-expressing cells, and in all cells, from which the values in non-expressing cells were also determined. Finally, the number of sub- and supranuclear transferrin vesicles per nucleus was calculated, and the ratio in transfected over non-transfected cells was determined.

Statistics

Data was visualized and statistical analyses were performed in GraphPad Prism (version 10.3.1). Gaussian distribution of the data was verified with multiple normality tests (D'Agostino & Pearson test, Anderson-Darling test, and Shapiro–Wilk test). To analyze statistical significance of two Gaussian distributed groups, an unpaired, two-tailed Student's t test was done. An F test was performed to test whether the groups had equal variance. If not, Welch's correction was applied. When comparing two groups without Gaussian distribution, a Mann-Whitney test was performed. When comparing multiple

normally distributed groups, a one-way ANOVA was used, followed by a Tukey's multiple comparisons test. For multiple groups without a normal distribution, a Kruskal-Wallis test, followed by a Dunn's multiple comparisons test was performed. For each experiment, the Figure legend states the number of replicates, the sample size, and the exact statistical test that was applied. In the graphs all statistically significant P-values ($P \leq 0.05$) are shown, as well as the mean ± standard error of the mean (SEM). When data is presented as SuperPlots, statistics were performed on the replicate means, shown as large symbols. The small symbols represent individual datapoints (images/meshworks/TALs) within these replicates.

Supplementary References

1. H. Gonschior *et al.*, Nanoscale segregation of channel and barrier claudins enables paracellular ion flux. *Nat. Comm.* **13**, 4985 (2022).
2. G. C. Ochoa *et al.*, A functional link between dynamin and the actin cytoskeleton at podosomes. *J. Cell Biol.* **150**, 377-389 (2000).
3. N. Blom, S. Gammeltoft, S. Brunak, Sequence and structure-based prediction of eukaryotic protein phosphorylation sites. *J. Mol. Biol.* **294**, 1351-1362 (1999).
4. M. Koval, Differential pathways of claudin oligomerization and integration into tight junctions. *Tissue Barriers* **1**, e24518 (2013).
5. F. A. Ran *et al.*, Genome engineering using the CRISPR-Cas9 system. *Nature Protocols* **8**, 2281-2308 (2013).
6. T. Otani *et al.*, Claudins and JAM-A coordinately regulate tight junction formation and epithelial polarity. *J. Cell Biol.* **218**, 3372-3396 (2019).
7. F. Bottanelli *et al.*, Two-colour live-cell nanoscale imaging of intracellular targets. *Nat. Comm.* **7**, 10778 (2016).
8. M. R. Beggs *et al.*, Claudin-2 and claudin-12 form independent, complementary pores required to maintain calcium homeostasis. *Proc Natl Acad Sci U S A* **118** (2021).
9. N. Himmerkus *et al.*, Calcium-Sensing Receptor in the Thick Ascending Limb and Renal Response to Hypercalcemia. *J Am Soc Nephrol* **36**, 1028-1039 (2025).
10. W. H. van Megen *et al.*, Gentamicin Inhibits Ca^{2+} Channel TRPV5 and Induces Calciuresis Independent of the Calcium-Sensing Receptor-Claudin-14 Pathway. *J Am Soc Nephrol* **33**, 547-564 (2022).
11. M. R. Beggs *et al.*, Expression of transcellular and paracellular calcium and magnesium transport proteins in renal and intestinal epithelia during lactation. *Am J Physiol Renal Physiol* **313**, F629-F640 (2017).
12. S. Frische *et al.*, Localization and regulation of claudin-14 in experimental models of hypercalcemia. *Am. J. Physiol. Renal Physiol.* **320**, F74-F86 (2021).
13. C. Prot-Bertoye *et al.*, Differential localization patterns of claudin 10, 16, and 19 in human, mouse, and rat renal tubular epithelia. *Am. J. Physiol. Renal Physiol.* **321**, F207-F224 (2021).
14. K. M. Kreusel, M. Fromm, J. D. Schulzke, U. Hegel, Cl^- secretion in epithelial monolayers of mucus-forming human colon cells (HT-29/B6). *Am. J. Physiol.* **261**, C574-C582 (1991).
15. D. Günzel *et al.*, Claudin-10 exists in six alternatively spliced isoforms that exhibit distinct localization and function. *J Cell Sci* **122**, 1507-1517 (2009).
16. I. Pouyiourou *et al.*, Ion permeability profiles of renal paracellular channel-forming claudins. *Acta Physiologica* **241**, e14264 (2025).
17. R. Greger, Cation selectivity of the isolated perfused cortical thick ascending limb of Henle's loop of rabbit kidney. *Pflügers Archiv* **390**, 30-37 (1981).
18. T. Breiderhoff *et al.*, Deletion of *claudin-10* (*Cldn10*) in the thick ascending limb impairs paracellular sodium permeability and leads to hypermagnesemia and nephrocalcinosis. *Proc. Natl. Acad. Sci. U.S.A.* **109**, 14241-14246 (2012).
19. A. Plain *et al.*, Corticomedullary difference in the effects of dietary Ca^{2+} on tight junction properties in thick ascending limbs of Henle's loop. *Pflugers Arch* **468**, 293-303 (2016).
20. L. L. Clarke, A guide to Ussing chamber studies of mouse intestine. *American Journal of Physiology-Gastrointestinal and Liver Physiology* **296**, G1151-G1166 (2009).
21. J. Schindelin *et al.*, Fiji: an open-source platform for biological-image analysis. *Nat. Methods* **9**, 676-682 (2012).
